# Metal-Free *N*-Doped Carbons for Solvent-Less CO_2_ Fixation Reactions: A Shrimp Shell Valorization Opportunity

**DOI:** 10.1021/acssuschemeng.2c04443

**Published:** 2022-09-29

**Authors:** Daniele Polidoro, Alvise Perosa, Enrique Rodríguez-Castellón, Patrizia Canton, Lidia Castoldi, Daily Rodríguez-Padrón, Maurizio Selva

**Affiliations:** † Dipartimento di Scienze Molecolari e Nanosistemi, Università Ca’ Foscari di Venezia, 30123 Venezia, Italy; ‡ Department of Inorganic Chemistry, Facultad de Ciencias, Universidad de Málaga, Campus de Teatinos s/n, 29071 Málaga, Spain; § Laboratory of Catalysis and Catalytic Processes, Dipartimento di Energia, Politecnico di Milano, Via La Masa 34, 20156 Milano, Italy

**Keywords:** metal-free, *N*-doped carbons, chitin, chitosan, shrimp shells, CO_2_ insertion, cyclic carbonates, continuous flow

## Abstract

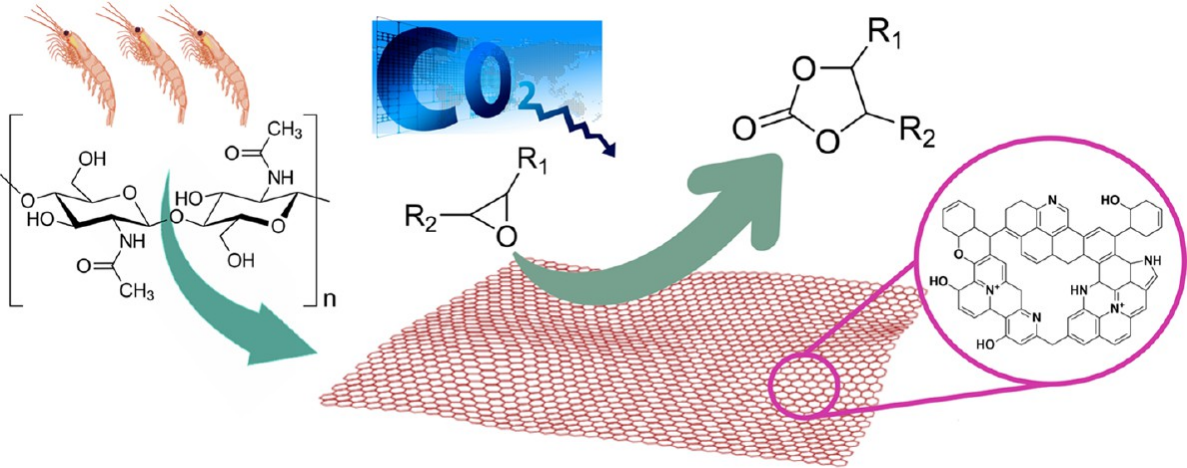

High anthropogenic CO_2_ emissions are among the main causes of climate change. Herein, we investigate the use of CO_2_ for the synthesis of organic cyclic carbonates on metal-free nitrogen-doped carbon catalysts obtained from chitosan, chitin, and shrimp shell wastes, both in batch and in continuous flow (CF). The catalysts were characterized by N_2_ physisorption, CO_2_-temperature-programmed desorption, X-ray photoelectron spectroscopy, scanning electron microscopy, and CNHS elemental analysis, and all reactivity tests were run in the absence of solvents. Under batch conditions, the catalyst obtained by calcination of chitin exhibited excellent performance in the conversion of epichlorohydrin (selected as a model epoxide), resulting in the corresponding cyclic carbonate with 96% selectivity at complete conversion, at 150 °C and 30 bar CO_2_, for 4 h. On the other hand, in a CF regime, a quantitative conversion and a carbonate selectivity >99% were achieved at 150 °C, by using the catalyst obtained from shrimp waste. Remarkably, the material displayed an outstanding stability over a reaction run time of 180 min. The robustness of the synthetized catalysts was confirmed by their good operational stability and reusability: ca. (75 ± 3)% of the initial conversion was achieved/retained by all systems, after six recycles. Also, additional batch experiments proved that the catalysts were successful on different terminal and internal epoxides.

## Abstract 2

Metal-free *N*-doped carbonaceous catalysts for the upgrading of CO_2_ toward cyclic carbonates under batch and continuous-flow regimes.

## Introduction

Climate change is already showing the detrimental effects that were predicted years ago, not only on the environment but also on our society, from health to the economic domains.^1,2^ The increase of global temperatures, CO_2_ concentration in the atmosphere, and sea levels worldwide are the most striking pieces of evidence, intrinsically related to the huge increase of anthropogenic activities and the boost of living standards still largely dependent on fossil fuels.^3,4^

Immediate actions are needed to limit and progressively remove all the causes responsible for this situation, among which moving toward a close CO_2_ cycle through biorefinery and circular economy concepts is included.^5−11^ Indeed, the chemical fixation of CO_2_, the most abundant and renewable carbon source on the planet, stands up as one of the best options to design innovative strategies for the synthesis and fabrication of intermediates, durable materials, and polymers with low carbon footprint.^12−15^

This approach has been proposed for a variety of substrates such as epoxides and aziridines to name a few, and it has been successfully applied to achieve high-added-value chemicals including cyclic carbonates, maleic anhydrides, benzoxazine-2-ones, oxazolidinediones, and 2-oxazolidinones, among others.^16−19^ Particularly relevant is the preparation of cyclic carbonates for the myriad of fields where these derivatives are involved in, from fuel additives, to lithium batteries as electrolytes, polar aprotic solvents, or feedstocks in the synthesis of polycarbonates.^20,21^

Both homogeneous and heterogeneous catalytic strategies have been described in recent years for the insertion of CO_2_ into epoxides. Beyond the use of (renewable) CO_2_, however, several other factors must be considered for the implementation of authentic sustainable transformations: among them, critical are the nature of the process itself and the catalytic systems, the approach and the chemicals employed for the synthesis of such catalysts, and their reusability and stability. For example, most of the methodologies so far employed for the synthesis of cyclic carbonates involved the use of either dangerous or costly/endangered metals, metal complexes, ionic liquids, metal-modified carbon nitride materials, metal organic frameworks (MOFs), organocatalysts based on alkylated or protonated bicyclic amidine or DBU (using halides as counteranions), and in several cases, the use of cocatalysts.^22^Table 1 summarizes some very recent results focused on the case of epichlorohydrin (ECl) as a model epoxide.^23^ The CO_2_ insertion on ECl has been investigated using catalysts based on cobalt complexes based on triazole, MOF-based materials (ZIF-8 integrated in a zirconosilicate zeolite), dendritic ionic liquids, a Zr-thiamine modified carbon nitride, and *N*-doped carbonaceous systems (entries 1–5). Notwithstanding the desired carbonate was achieved in good-to-nearly quantitative yields under relatively mild conditions, any of these protocols shows some environmental and synthetic issues including the expensive catalyst preparation, the use of additional reagents/promoters, more often in the form of toxic onium salts or strong acids,^20,24,25^ long reaction times (up to 5 days),^26^ and extra (intercalation) approaches to support ionic liquids on solid materials.^27^

**Table 1:** Comparative Results Reported in the Literature for the Insertion of CO_2_ on Epichlorohydrin (EPI)

entry	catalyst	conditions	conversion	selectivity	ref
1	triazole-cobalt(II) complex	1 bar/120 °C/5 h	100	97	(20)
2	ZIF-8-nano-ZrS	10 bar/120 °C/2 h	96	95	(26)
3	dendritic ionic liquid	150 bar/130 °C/2.5 h	90	99	(27)
4	Zr-thiamine-modified carbon nitride	20 bar/120 °C/6 h	95	97	(24)
5	*N*-doped carbon	15 bar/150 °C/15 h	99	99	(25)
6	chitin-derived *N*-doped carbon	30 bar/150 °C/4 h	99	94	this work
7^a^	chitin-derived *N*-doped carbon	150 °C	75	95	this work
		0.01 mL/min			
8^a^	shrimp shell-derived *N*-doped carbon	150 °C	99	99	this work
		0.01 mL/min			

a Experiments performed under continuous-flow (CF) conditions.

In this context, simplicity emerges as a fundamental need where the catalyst design should be integrated with biomass/biowaste valorization, under the umbrella of the green chemistry principles and the circular economy. This paper has been focused on this objective by exploring fishery waste as a secondary raw material which is not only largely available, but it displays an extraordinary chemical richness worthy of valorization for chemical/catalyst preparation.^28,29^

Chitin, the second most abundant biopolymer on the planet after cellulose, is relevant in this area for the fabrication of *N*-doped carbonaceous solids including, for example, catalysts.^30,31^ Although biocompatibility, biodegradability, and nontoxicity are recognized strengths of chitin, its insolubility in all common solvents is an issue for practical applications. A solution to this problem is often in the use of chitosan, which derives from the partial deacetylation of chitin, via enzymatic treatments, steam explosion, or alkali processing. Chitosan preserves most of the properties of chitin but, unlike the latter, it is somewhat soluble in acidic aqueous solutions. Several C/N-based materials have been synthesized from chitosan; particularly, catalysts for CO_2_ cycloaddition were recently achieved through a preactivation step with phosphoric acid followed by a thermal treatment.^25^

In this work, shrimp shells (SSs) have been considered as a source of chitin suitable to synthesize *N*-doped carbonaceous materials with built-in basic properties.^32,33^ A procedure to convert SSs or chitin and chitosan into slightly basic solids has been coupled to the use of such materials as heterogenous catalysts for the CO_2_ fixation into epoxides. A total of five samples have been obtained, all of which demonstrated significant activities for the desired reaction: at complete conversion of the starting epoxide, a selectivity up to 95% was achieved toward the corresponding cyclic carbonate. Moreover, as a further evolution of this study, the CO_2_ cycloaddition was implemented in the CF mode. To the best of our knowledge, only a few examples have been reported for the CF synthesis of cyclic carbonates from epoxides, among which there is a recent study performed by our group based on a binary catalytic mixture composed of diethylene glycol/NaBr.^34^ The use of *N*-doped carbonaceous materials derived from fish waste and chitin/chitosan allowed a robust CF design with a productivity up to 15 mmol g^–1^ h^–1^ amenable for medium-to-large scale productions of highly pure cyclic carbonates.

## Experimental Section

### Materials and Equipment

EPI, epibromohydrin, glycidol, 1,2-epoxybutane, 1,2-epoxyhexane, cyclohexene oxide, styrene oxide, glycidyl propargyl ether, limonene oxide, acetonitrile, methanol, phosphoric acid, chitin, and chitosan were commercially available compounds sourced from Sigma-Aldrich. If not otherwise specified, reagents and solvents were employed without further purification. Shrimp wastes were obtained from a local fish market. Water was Milli-Q grade. CO_2_ gas was purchased from SIAD, Italy. Gas chromatography–mass spectrometry (GC–MS) (EI, 70 eV) analyses were performed on a HP5-MS capillary column (*L* = 30 m20, Ø = 0.32 mm, film = 0.25 mm). GC (flame ionization detector; FID) analyses were performed with an Elite-624 capillary column (*L* = 30 m, Ø = 0.32 mm, film = 1.8 mm). ^1^H and ^13^C NMR spectra were recorded in the Bruker Advance III HD 400 WB equipped with a 4 mm CP/MAS probe, at 400 and 101 MHz, respectively. Chemical shifts were reported downfield from tetramethylsilane, and MeOD was used as a solvent. All reactions were performed in duplicate to verify reproducibility.

### Synthesis of N-Doped Carbonaceous Materials

In a typical synthesis, 10 g of biomass (chitin, chitosan, or shrimp wastes) were heated at 450 °C (heating rate was 1 °C/min) under N_2_ flow (10 mL min^–1^) for 1 h. For C1-500 and C3-500, the starting biomass-derived compounds (chitosan and chitin, respectively) were mixed with 50 wt % aqueous phosphoric acid solution in a 1:2 ratio and aged for 24 h at room temperature, while for C2-500, C4-500, and C5-500 (chitosan, chitin, and shrimp wastes, respectively) no pretreatment was carried out, and the starting materials were used as received. The resulting carbons were ground to powder (particle size <200 μm) and stored in an oven (60 °C, 15 mbar) until further use. The yield of carbon materials was 20 ± 5%.

## Material Characterization

The analysis of the chemical composition on the surface of the solids was carried out by X-ray photoelectron spectroscopy (XPS) with a Physical Electronics VersaProbe II Scanning XPS Microprobe equipped with monochromatic X-ray Al Kα radiation at a vacuum of 10^–7^ Pa. The binding energies were referenced to the C 1s peak from adventitious carbon at 284.8 eV. High-resolution spectra were recorded using a concentric hemispherical analyzer in a 29.35 eV constant energy pass, using a 200 μm diameter analysis area, and the pressure in the analysis chamber was kept below 5 × 10^–6^ Pa. PHI ACCESS ESCA-FV6 software was used for data acquisition and analysis. A Shirley-type background was subtracted from the signals. The recorded spectra were always analyzed with Gauss–Lorentz curves in order to determine more accurately the binding energy of the atomic levels of the different elements.

Concentrations of basic sites on carbon samples were determined by temperature-programmed desorption (TPD). For basicity measurements, TPD using CO_2_ (8000 ppm in He) as a probe was used. In a typical measurement, 60 mg of sample was placed in a tubular quartz reactor (d.i.7 mm) and evacuated at 150 °C for 2 h under He flow. Then the sample was cooled to 50 °C in He flow. At this temperature, CO_2_ was passed through the sample for 1 h followed by heating to 80 °C in He flow for 1 h in order to remove physisorbed CO_2_. TPD was carried out from 50 to 350 °C at a heating rate of 10 °C/min under He flow. The gas analysis has been performed by online micro-GC and mass spectrometry.

N_2_ physisorption measurements were conducted on a Micromeritics TriStar 3000 instrument. The samples were outgassed at 120 °C for 2 h. Then, adsorption–desorption isotherms were recorded at −196 °C. The specific surface areas were calculated by the Brunauer–Emmett-Teller (BET) method; the pore volumes were calculated from adsorption isotherms, and the pore size distributions were estimated using the Barrett, Joyner, and Halenda (BJH) algorithm available as a built-in software from Micromeritics.

Scanning electron microscopy (SEM) measurements and energy-dispersive X-ray spectrometry (EDS) analysis were carried out with a Carl Zeiss Sigma VP field emission scanning electron microscope (FE-SEM) equipped with a Bruker Quantax 200 microanalysis detector. The EDS spectra and maps were recorded under the same conditions (20 keV) for all the samples.

## Catalytic Experiments

### General Procedures for CO_2_ Insertion into Epoxides under Batch Conditions

The selected epoxide (20 mmol) and catalyst (100 mg) were charged in a round-bottomed flask shaped as a test tube, which was equipped with a pierced glass cap and a stirring bar. The flask was placed inside a 100 mL stainless-steel autoclave, which was sealed, degassed via three vacuum-CO_2_ cycles, pressurized with CO_2_ (5–30 bar), and finally heated at *T* of 25–150 °C for 2–15 h. Thereafter, the autoclave was cooled to room temperature and vented. Upon completion of reaction, the product from the autoclave was diluted with methanol (5 mL) and analyzed by GC-FID, GC–MS, and NMR. All characterization data are reported in the ESI section (Figures S1–S14).

### General Procedures for CO_2_ Insertion into Epoxides under CF Conditions

In a typical procedure, the CF apparatus was first conditioned with acetonitrile (*F* = 0.5 mL·min^–1^) and CO_2_ (*F*_CO2_ = 4 mL·min^–1^) for 30 min. Then, a homogeneous 0.3 M solution of EPI in acetonitrile or neat EPI was continuously pumped to the CF reactor at the desired *T* and flow rates (*T* = 200–150 °C, *F* = 0.3–0.01 mL·min^–1^, and *F*_CO2_ = 1 mL·min^–1^). Reaction runs were conducted for 120 min, though some prolonged tests were carried out for up to 200 min. The reaction mixture was collected and analyzed by GC-FID to determine the conversion and selectivity. After each test, the CF system was washed with acetonitrile (100 mL) and dried with a CO_2_ flow (*F*_CO2_ = 3 mL·min^–1^) for 10 min.

## Results and Discussion

### Catalyst Preparation

**Figure 1:**
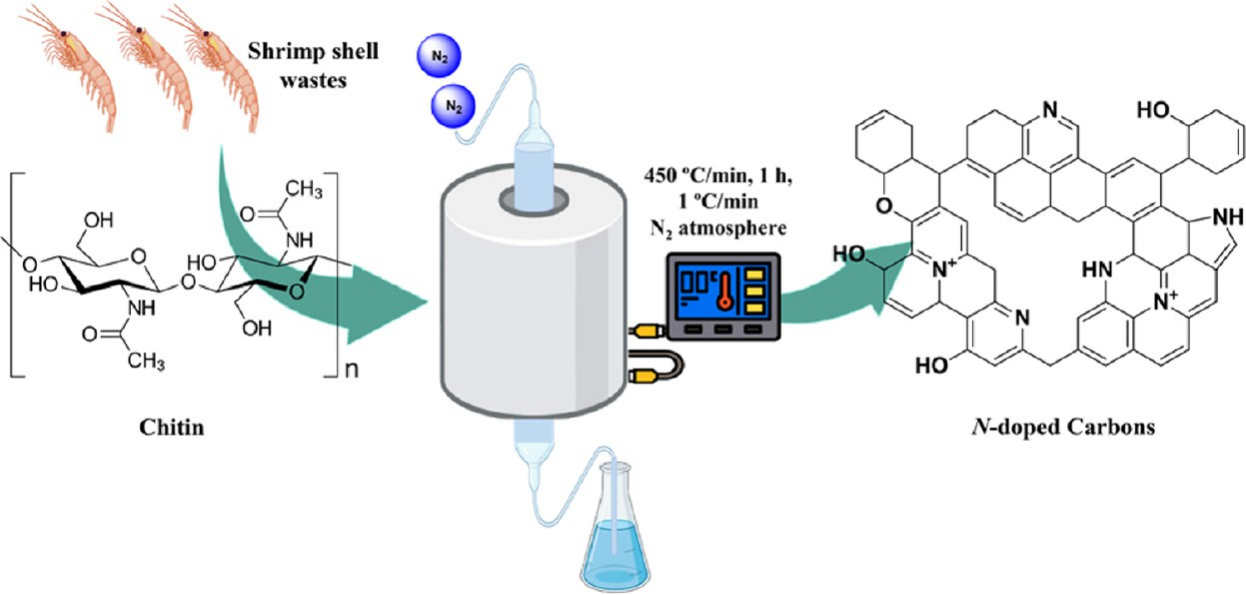
General procedure employed for the synthesis of *N*-doped carbons from chitin, chitosan, or SS wastes.

The literature describes a variety of procedures for the preparation of *N*-doped materials from either fish (crustacean) biowaste or N-containing polysaccharides, in particular chitosan and to a lower extent, chitin.^30,31,35^ The same three feedstocks, chitosan, chitin, and SS residues, were used in this work to synthesize a series of *N*-doped carbons. With respect to already reported methods,^25^ the preparation protocol was designed as simple/sustainable as possible, that is, by simply heating the starting solid (chitosan, chitin, SS) at 450 °C in an inert atmosphere, in the absence of additional reagents. In some cases, however, a pretreatment with phosphoric acid was considered to compare the effect on the catalyst performance. Figure 1 and Table 2 summarize the salient aspects of the synthesis and the achieved catalysts.

**Table 2:** Catalysts Used in This Work

entry	starting material	preparation method	catalyst label
1	chitosan	aging with H_3_PO_4_ (50 wt %)-pyrolysis	C1-500
2	chitin	aging with H_3_PO_4_ (50 wt %)-pyrolysis	C2-500
3	chitosan	pyrolysis	C3-500
4	chitin	pyrolysis	C4-500
5	shrimp shell	pyrolysis	C5-500

### Catalytic Activity

EPI, which is one of the most investigated substrates for the CO_2_ insertion reactions, was selected in this study as a model epoxide to investigate the catalytic performance of the prepared materials. Batch experiments were carried out in a stainless-steel autoclave in which a mixture of EPI (20 mmol) and the catalyst (100 mg) was set to react at 150 °C under 30 bar of CO_2_ for 15 h. For comparison, additional tests were performed without any catalyst, and with commercial samples of an active carbon material (Activated Charcoal Norit, CAS: 7440-44-0, Merck), chitin, and chitosan, respectively.

**Scheme 1:**
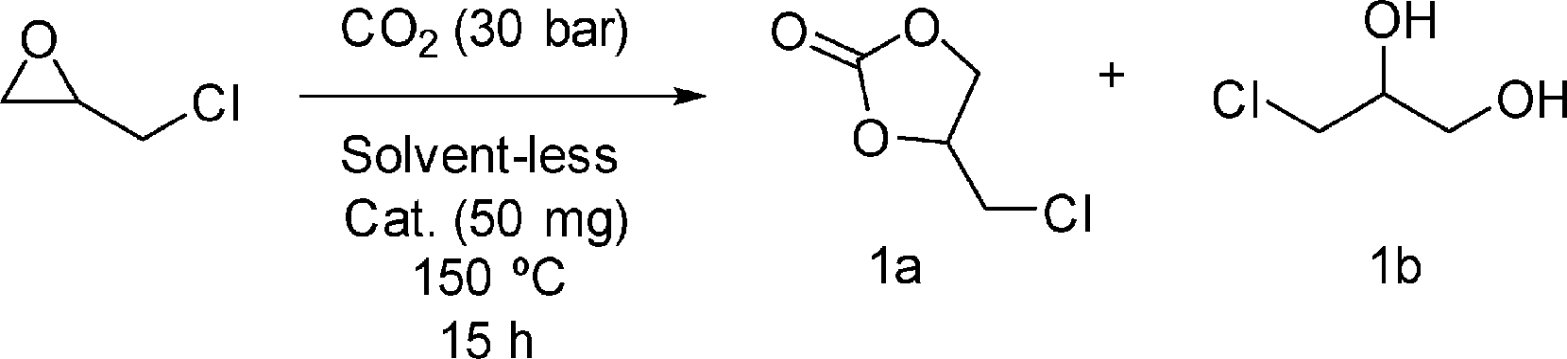
CO_2_ Insertion Reaction into EPI

NMR and GC/MS analyses of the reaction mixture confirmed the formation of the desired carbonate [4-(chloromethyl)-1,3-dioxolan-2-one, 1a] along with minor amounts of 3-chloropropane-1,2-diol (1b) (Scheme 1).

**Figure 2:**
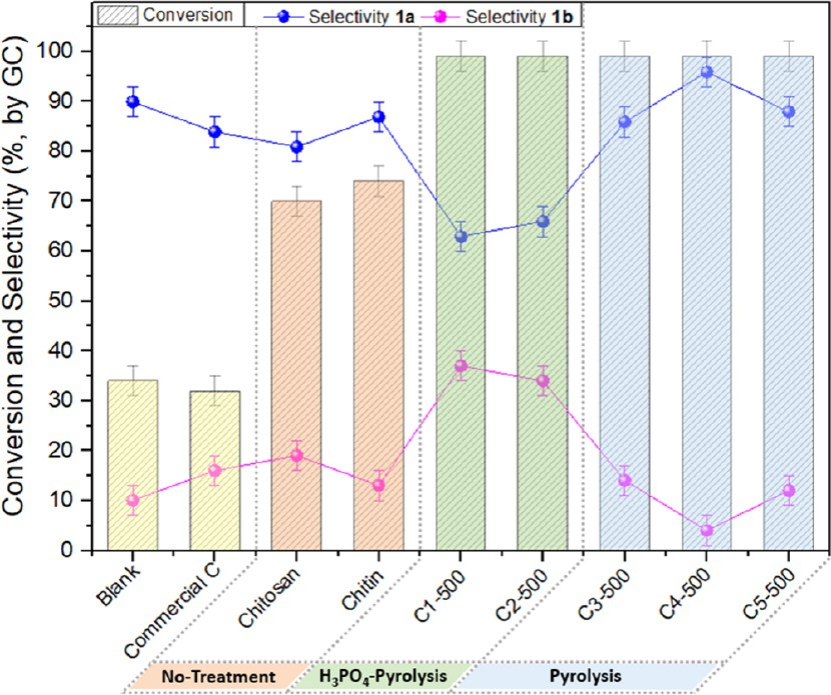
Catalyst screening in the CO_2_ insertion reaction into EPI. Reaction conditions: EPI (10 mmol); catalyst amount: 50 mg; *t*: 15 h; *T*: 150 °C, CO_2_ pressure: 30 bar. Conversion and selectivity were determined by GC.

Figure 2 reports the results by showing the conversion of EPI and the selectivity toward 1a and 1b, respectively.

The cycloaddition of CO_2_ occurred even without any catalyst (blank test): albeit the selectivity to 1a was remarkable (90%), a poor conversion was reached, not exceeding 34%. The formation of the diol 1b as a side-product was imputed to traces of water that could not be removed from the reaction environment, most plausibly associated with EPI, which is known to be a hygroscopic compound.^36^

Overall, the high electrophilic reactivity of EPI was apparently responsible for both a thermal insertion of CO_2_ and to a lower extent, a hydrolytic aperture yielding 1b. A similar result was achieved when the reaction was run in the presence of a commercial carbon, thereby confirming that this solid material did not possess any catalytic activity toward the desired cycloaddition process.

A quite different behavior was instead noticed by using commercial chitosan and chitin as received (without further treatments). Both biopolymers allowed doubling the conversion of EPI up to 70–74% compared to the noncatalyzed reaction. Such an outcome was ascribed to the basic properties of chitosan and chitin, different from those of natural polysaccharides such as cellulose or pectin, which are typically either acidic or neutral.^37^ Notwithstanding the encouraging activity, the carbonate selectivity was moderate (81–84%), and the use of chitin and chitosan was limited by the deactivation of such solids. Recycling tests—not reported here—confirmed that both biopolymers were no longer effective after a single reaction. A far better performance was achieved with *N*-doped carbocatalysts C*n*-500 (*n* = 1–5; Table 2 and Figure 2). Regardless of the starting feedstock, chitin, or chitosan, from which they were prepared, all the systems allowed a quantitative conversion of EPI. Remarkable differences, however, emerged by comparing the product distribution. In the case of C1-500 and C2-500 obtained by a phosphoric acid pretreatment, the selectivity toward the carbonate 1a was rather low, 63 and 66%, respectively. On the contrary, the 1a selectivity greatly improved when nonacid treated catalysts were used, from 86–88% up to an excellent value of 96% for C3/C5-500 and C4-500 solids, respectively. These results led us to conclude that the calcination of the starting materials was crucial to achieve active catalysts from chitin or chitin-derived biopolymers, but to make these materials selective for the CO_2_ insertion into epoxides, it was equally critical to control the pretreatment of such solids and avoid acid usage.

Even with *N*-doped carbocatalysts, the hydrolytic ring-opening of EPI to the diol 1b was the only competitive process to the CO_2_ cycloaddition. Additional experiments confirmed that under the conditions of Figure 2, but in the absence of CO_2_, the diol 1b was the almost exclusive derivative (ESI Section, Table S1). This side-reaction was never completely ruled out, though it was almost suppressed in the presence of C4-500 as a catalyst. The result suggested that if the CO_2_ insertion was rapid enough to produce the carbonate product 1a, the latter was substantially stable to hydrolysis and did not further react to originate the side-product 1b.

The best performant and most selective catalyst, C4-500, was selected to continue the study through a parametric analysis and the investigation of the substrate scope, followed by CF experiments. These will be detailed in the following sections.

### Influence of Reaction Parameters

**Figure 3:**
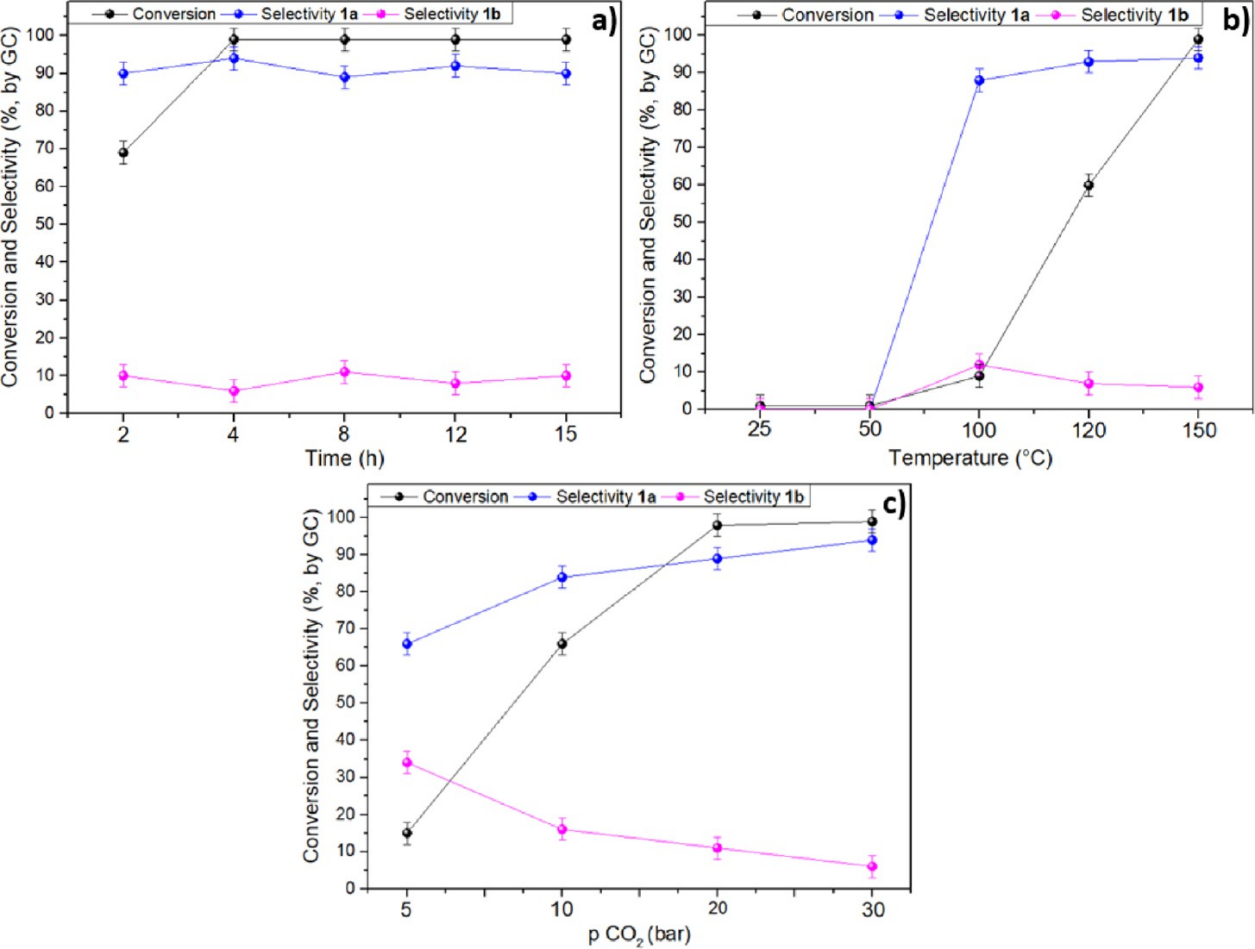
Influence of major reaction parameters on the catalytic performance of the *N*-doped catalysts, considering (a) time, (b) temperature, and (c) CO_2_ pressure.

The effect of major reaction parameters such as time (*t*), temperature (*T*), and CO_2_ pressure (*p*) was investigated by performing three sets of tests during which the cycloaddition of CO_2_ to EPI was carried out by changing: (i) *t* from 2 to 15 h, at 150 °C and 30 bar; (ii) *T* from 25 to 150 °C, at 30 bar for 4 h; and (iii) *p* from 5 to 30 bar, at 150 °C for 4 h. Results are shown in Figure 3a–c, respectively.

At 150 °C and 30 bar, the conversion increased from ca. 70 to >99% by increasing the reaction time 2 to 4 h (Figure 3a), while the selectivity toward the cyclic carbonate was steady at 90–92%. No changes of both the conversion (quantitative) and the product distribution (1a: 92 ± 3%) were appreciated by further prolonging the reaction. Subsequent experiments were therefore carried out for 4 h.

The temperature had a dramatic influence on the reaction rate (Figure 3b). At 30 bar and after 4 h, neither the cycloaddition of CO_2_ nor the hydrolysis of the EPI was observed below 100 °C. Even at this *T*, however, the conversion was moderate, not exceeding 9%. A substantial improvement of the reaction outcome was noticed at 120 °C and finally at 150 °C where the conversion gradually increased from 60 to >99%, respectively, with a carbonate selectivity of ca. 92%.

Finally, the effect of the CO_2_ pressure was investigated (Figure 3c). At 150 °C and after 4 h, both conversion and 1a selectivity improved from 15, to 66, 98, and >99%, and from 66, 84, 89, and 94%, respectively, when the pressure was increased from 5 to 30 bar. This trend reflected the progressively larger availability of CO_2_ in the reaction mixture for the formation of the carbonate.

Overall, based on these results, the best reaction conditions were found at 150 °C, 30 bar, and 4 h.

**Scheme 2:**
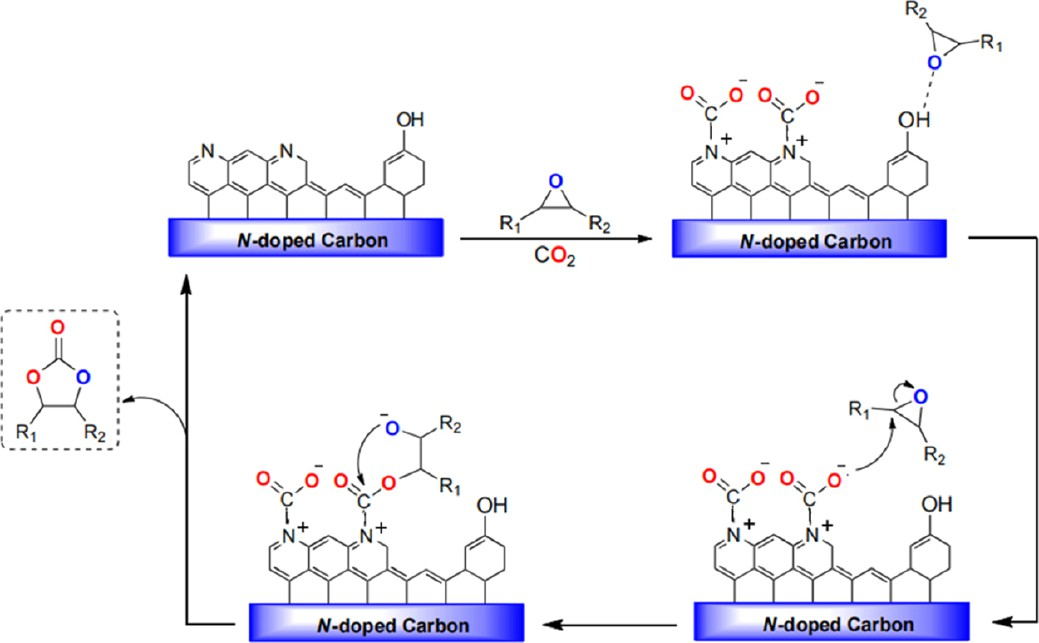
Proposed Mechanism for the *N*-Doped Carbon-Catalyzed CO_2_ Insertion Reaction into Epoxides

Based on the experimental results, a plausible mechanism is proposed through a Lewis-base catalyzed pathway (Scheme 2). The reaction likely proceeds through the initial adsorption/activation of CO_2_ and the epoxide on the Lewis basic site (pyridinic and the pyrrolic N centers) and on the oxygen functionalities (−OH and −COOH), respectively. Subsequently, the epoxide is desorbed, while CO_2_, through an intramolecular nucleophilic attack, is inserted into the C–O bond of the epoxide, leading to the formation of the carbonate product.

### Catalyst Reusability

**Figure 4:**
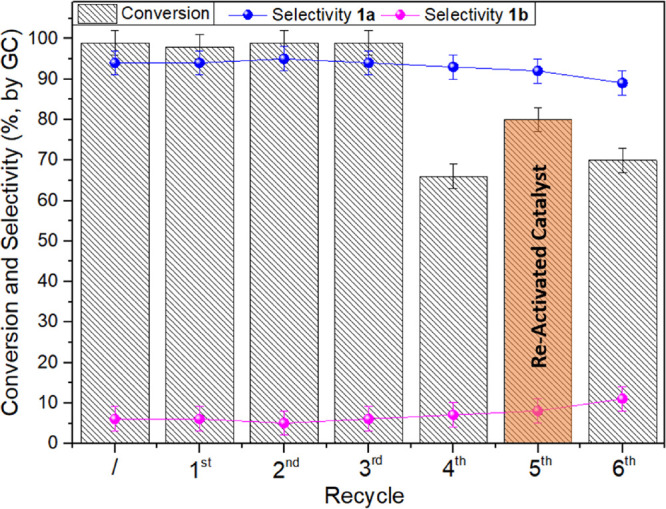
Catalyst reusability study. Conditions for each run: EPI (20 mmol), C4-500 (100 mg) at 150 °C under 30 bar of CO_2_ for 4 h. After the fourth recycle, the catalyst was re-activated by thermal treatment at 180 °C, under N_2_ flow for 18 h.

The stability and reusability of the catalyst C4-500 were investigated by designing recycling experiments under the above-described optimized conditions. Once a first reaction was complete, the catalyst was filtered and washed with methanol (30 mL) and dried overnight. The recovered catalyst was added with fresh EPI (10 mmol, 925 mg), and a new reaction was started. This sequence was repeated seven times, and each reaction was run twice to ensure reproducibility. All experiments were run at 150 °C, 30 bar, and for 4 h. The results are reported in Figure 4. Both conversion and 1a selectivity were steady at >99% and 94–95%, respectively, during the initial four runs. A remarkable drop of activity was, however, observed in the fifth run (fourth recycle: conversion 66%). After this test, the catalyst was filtered, dried, and heated at 180 °C under a nitrogen flow (10 mL min^–1^) for 18 h. The thermal treatment allowed a partial recovery of the catalytic performance: indeed, in a subsequent recycling experiment, an 80% conversion was reached with 92% selectivity toward 1a (sixth run, fifth recycle). A final recycling test proved that the C4-500 sample was undergoing a progressive deactivation that caused either a decrease of the reaction rate and a slight, but detectable, decrease in the formation of the carbonate (7th run: conversion and selectivity of 70 and 89%, respectively). The mechanism and reasons for this behavior were investigated by analyzing structural and chemical changes on the surface of the catalytic materials after the reaction/reaction cycles. This was carried out through a comprehensive approach based on an array of techniques described in the following paragraphs.

### Characterization Analyses

The catalytic materials were characterized by a multitechnique approach, involving CNHS elemental analysis, XPS, N_2_-physisorption measurements, CO_2_-TPD, and SEM. For this purpose, the samples C4-500 and C5-500 were chosen as the most representative materials of this study. A specimen of C4-500 was characterized also after its use: the catalyst was recycled four times as described in Figure 3, and then it was carefully washed with methanol and dried at 100 °C for 24 h. The recovered material was labeled as C4-500-used.

### Elemental Analyses

A first relevant aspect was the evaluation of the nitrogen content of the investigated materials because this could be associated with the basic properties of the solids (Figure 1), and in the last analysis, with their catalytic performance. CNHS measurements indicated that the nitrogen content was (10 ± 1)% and (6 ± 1)% for C4-500 and C5-500, respectively.

To inspect these results more closely, further CNHS analyses were carried out on other *N*-doped carbons of Table 2. It was demonstrated that samples subjected to a phosphoric acid pretreatment (C1-500 and C3-500) displayed a lower N content of (4 ± 1)%, while the nontreated C2-500 exhibited an N-loading of (10 ± 1)% comparable to that of the most active system C4-500.

Albeit phosphoric acid has been widely employed as a dehydrating agent for the chemical activation of carbonaceous samples; its use was apparently detrimental on the synthesis of catalysts studied here, most likely because of the concomitant formation of P- and N-combined derivatives. Moreover, some studies in the literature have also proposed that, besides decreasing in N content, the use of phosphoric acid could cause the inactivation of pyridinic-N sites both by protonation (lowering the density of these active sites) and a site blocking effect via adsorption of the phosphate ions.^38^

Finally, compared to the fresh sample, also C4-500-used showed a lesser nitrogen loading (4 ± 1%). It was postulated that under the explored reaction conditions, an adsorption/coordination of organic species over the catalyst surface was responsible for the N-loss.

### N_2_-Physisorption and Surface Basicity

**Figure 5:**
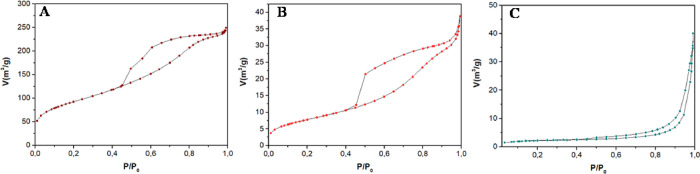
N_2_-physisorption isotherms of C4-500 (A), C4-500-used (B), and C5-500 (C) samples.

The textural properties of C4-500, C5-500, and C4-500-used samples were investigated by N_2_-physisorption analysis. The obtained isotherms are displayed in Figure 5.

Both the fresh sample and the reused sample of C4-500 exhibited a type IV isotherm (Figure 5A,B), characteristic of mesoporous materials, with adsorption hysteresis type II, associated with disordered networks. In turn, the C5-500 sample still showed a type IV isotherm but with hysteresis type III (Figure 5C), indicating the formation of mesoporous materials with nonrigid particle aggregates.^39^ The textural properties, namely, surface area, pore diameter, and pore volume, are reported in Table 3.

**Table 3:** Characterization Analyses of the *N*-Doped Carbonaceous Materials

material	*S*_BET_ [m^2^/g]^a^	*D*_BJH_ (nm)^b^	*V*_BJH_ [cm^3^/g]^c^	CO_2_-TPD [μmol/g]
C4-500	321	4.5	0.38	268.9
C4-500-used	29	6.8	0.06	743.5
C5-500	7	26.8	0.06	464.4

a *S*_BET_: specific surface area was calculated by the BET equation.

b *D*_BJH_: mean pore size diameter was calculated by the BJH equation.

c *V*_BJH_: pore volumes were calculated by the BJH equation.

The C4-500 sample displayed a good surface area of 321 m^2^/g with a mean pore diameter of 4.5 nm and a pore volume of 0.38 cm^3^/g (entry 1). These properties, however, drastically changed for the used catalyst (C4-500-used) that showed a decrease of surface area down to 29 m^2^/g, a critical reduction of the pore volume (0.06 cm^3^/g), and an increment of the mean pore diameter up to 6.8 nm (entry 2). Overall, this was consistent with the occurrence of occlusion phenomena because of the adsorption of organic moieties under the reaction conditions. Smaller pores were therefore clogged leading to a shift of the pore diameter distribution toward higher values.

A low surface area (7 m^2^/g) was measured for C5-500, coherently with the complex precursor matrix (SS) used to prepare the catalyst.^40^ Compared to C4-500, the sample displayed a higher mean pore diameter of 26.8 nm which was still within the range of mesoporous solids (entry 3).

Table 3 also reports the surface basicity of C4-500, C5-500, and C4-500-used samples, determined by TPD using CO_2_ as a probe. Values were comparable to those previously reported in the literature for *N*-doped carbons, thereby confirming the suitability of the prepared materials as catalysts for the investigated reaction.^25^ Interestingly, the SS-derived solid showed a basicity almost 50% higher than the sample obtained from chitin (entries 1 and 3). This suggested that the presence of proteins and/or other mineral components in the starting raw material (SS) had a positive influence on the concentration of basic sites. However, if, on the one hand, basic functionalities were crucial for the progress of the cycloaddition reaction, they could also promote the irreversible poisoning of the catalyst surface. This probably occurred in the case of the C4-500-used sample whose surface basicity was unexpectedly high, more than 2.5 higher than that of the original fresh C4-500. Indeed, in agreement with other literature analyses,^25^ the poisoning of the catalyst was ascribed to the chemisorption of organic compounds bearing oxygenated (basic) groups as the starting epoxide, the cyclic carbonate, and/or their derivatives.

### SEM–EDX Analyses

**Figure 6:**
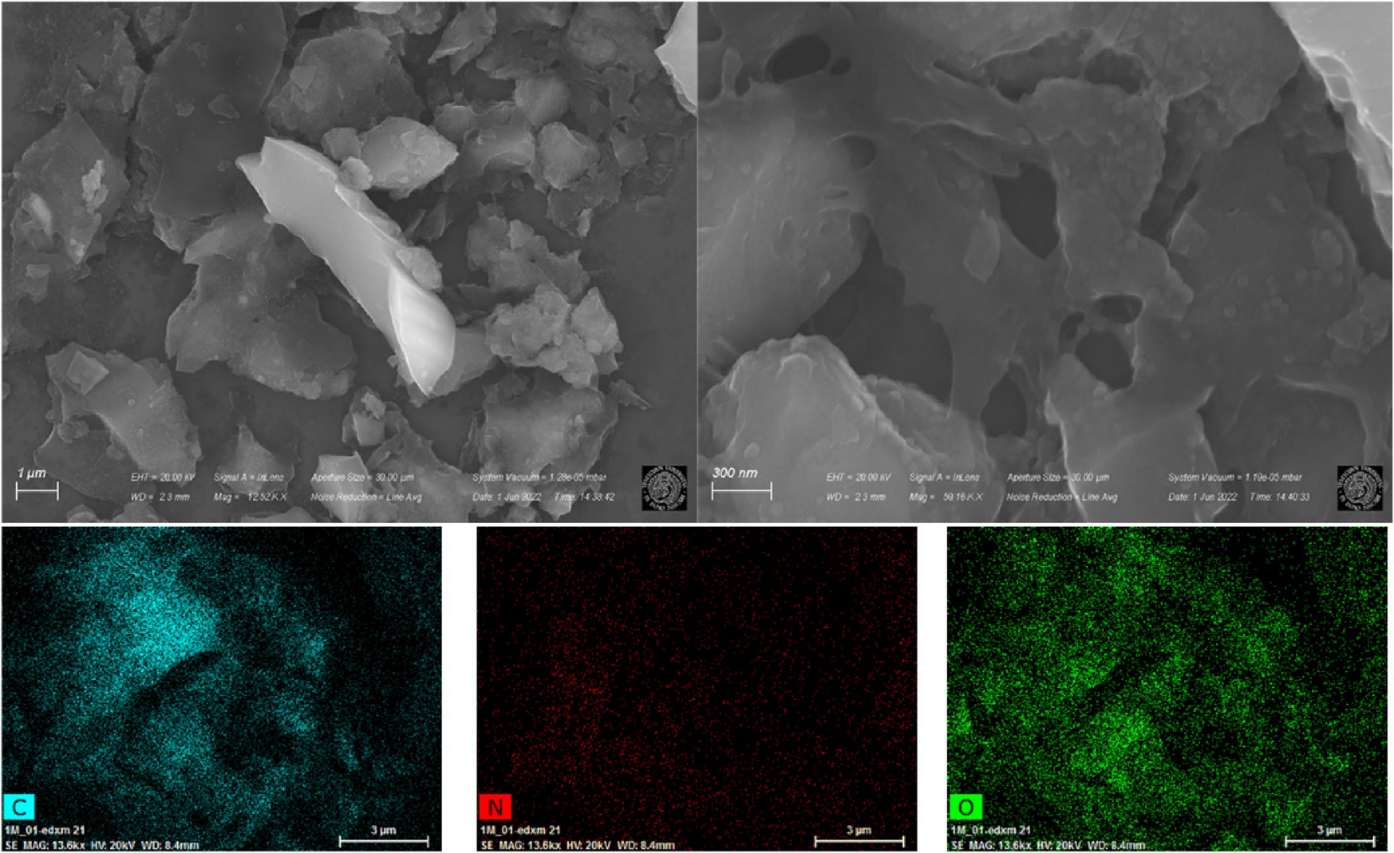
SEM micrographs and SEM-mapping analysis of the C4-500 catalytic material.

**Figure 7:**
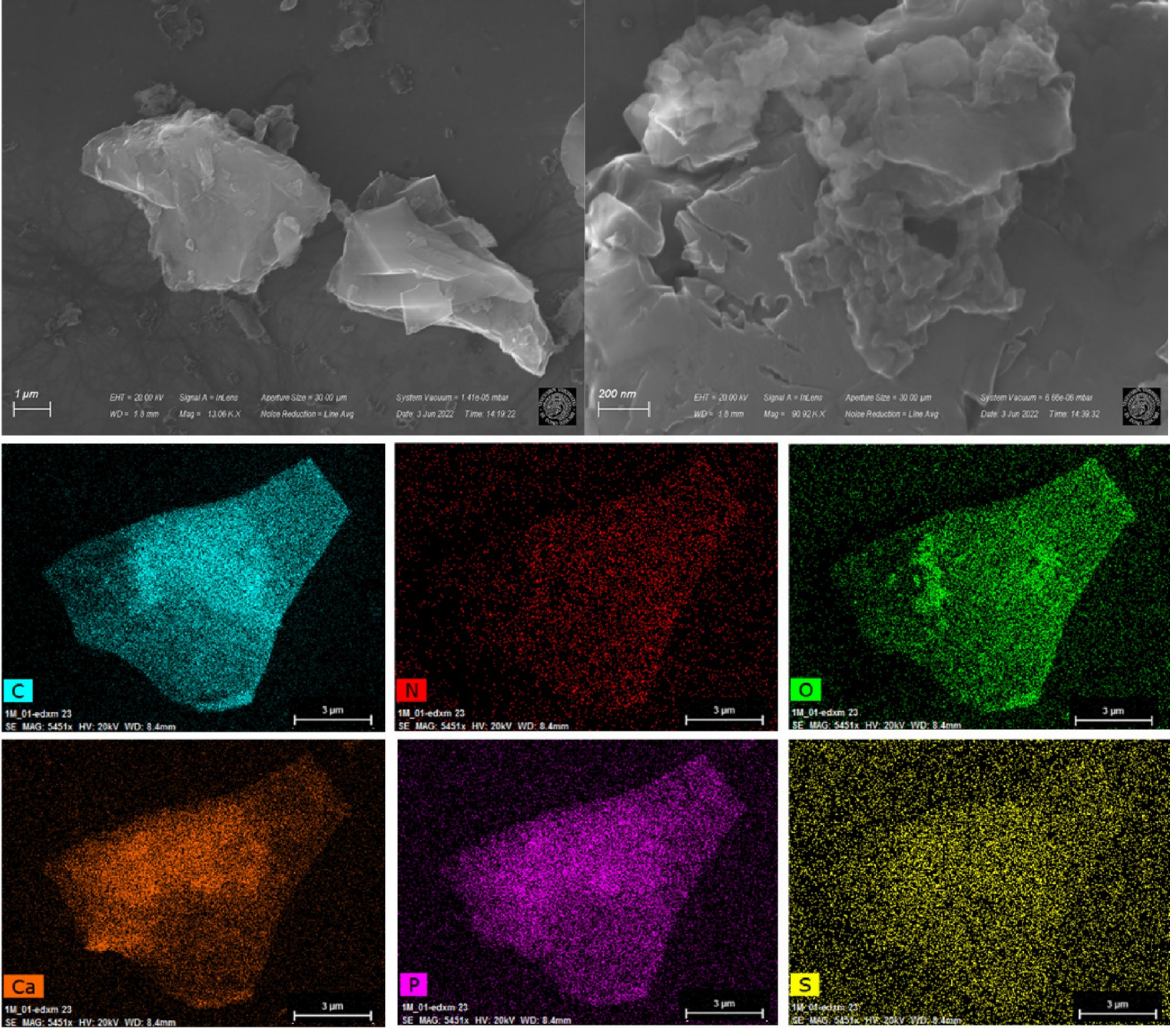
SEM micrographs and SEM-mapping analysis of the C5-500 catalytic material.

SEM micrographs of C4-500 and C5-500 are shown in Figures 6 and 7, respectively.

The presence of agglomerated particles with the formation of a highly porous network involving both intra and interparticle porous materials was inferred from the SEM images of C4-500.^41^ Such observation was consistent with the results of N_2_ physisorption. Moreover, the SEM-mapping of the sample revealed a homogeneous distribution of the constituent elements (C, O, N) of the catalyst surface, with nitrogen in a lower concentration compared to carbon and oxygen, as suggested also from CNHS analyses.

SEM images of the C5-500 sample showed the presence of a laminar structure where various *N*-doped carbon layers looked overlayed with each other. Even if to a lower degree with respect to C4-500, a certain porous architecture was observed also in this case. The SEM-mapping results indicated not only the presence of carbon, nitrogen, and oxygen, but also calcium, phosphorous, and sulfur, that were derived from the starting precursor (SSs). The presence of nitrogen was homogeneous over the surface of the sample, thereby implying the availability of the element in the main (basic) active sites for the catalytic process.

### XPS Analyses

The chemical nature and the elemental composition on the surface of the C4-500, C5-500, and C4-500-used samples were determined by XPS and are reported in Figure 7.

**Figure 8:**
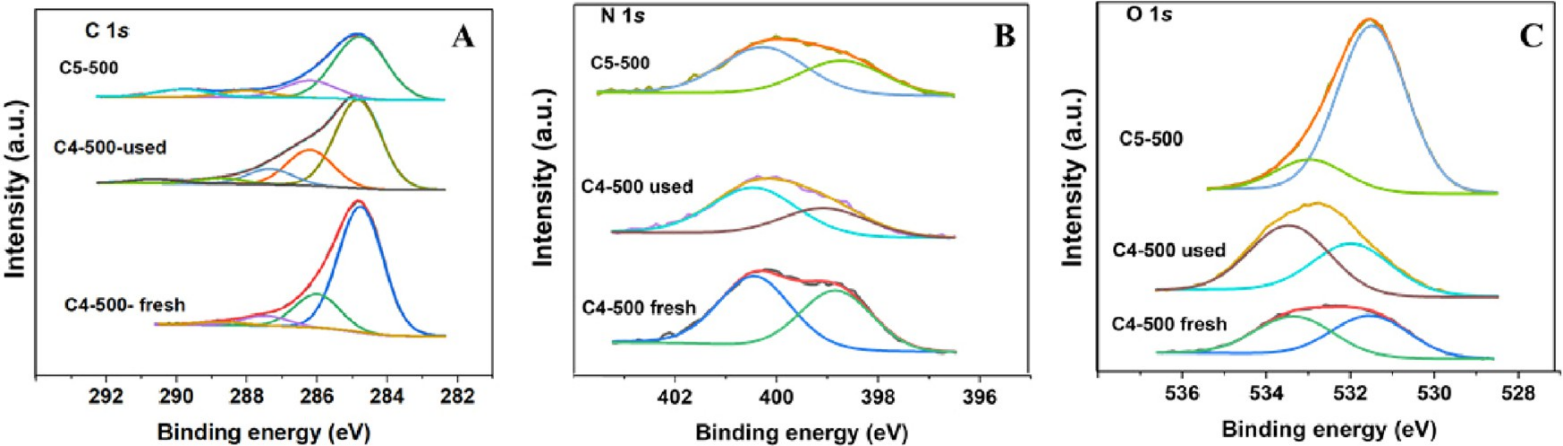
XPS spectra of C4-500, C5-500, and C4-500-used samples in (A) C *1s* region, (B) N *1s*, and (C) O *1s* regions.

The presence of carbon, oxygen, and nitrogen was confirmed for all three catalysts. In particular, the C *1s* XPS region of the C4-500 sample displayed the presence of four contributions located around 284.8, 286.0, 287.5, and 288.8 eV, attributed to C–C/C=C (graphitic, aromatic carbon), C–OH, C–N/C–O, and C=O bonds, respectively (Figure 8a). Similarly, the C5-500 material showed the presence of four signals at 284.8 eV (C–C/C=C), 286.2 eV (C–OH), 288.0 eV (C–N/C–O), and 289.8 eV (C=O). In both cases (C4-500 and C5-500), XPS quantification analysis of the components in the C *1s* XPS region revealed a contribution of the C–N/C–O of ca. (7 ± 1)%. The XPS analysis of the C4-500-used sample in the C *1s* region showed the presence an additional signal (besides the ones previously observed in the fresh C4-500 sample) located at 290.6 eV (Figure 8b). This was attributed to COO^–^ (carboxylate) bonds,^42^ and it was most likely due to the partial adsorption of carbonate products on the catalyst surface, in agreement with N_2_-physisorption, CO_2_-TPD, and CNHS measurements.

**Figure 9:**
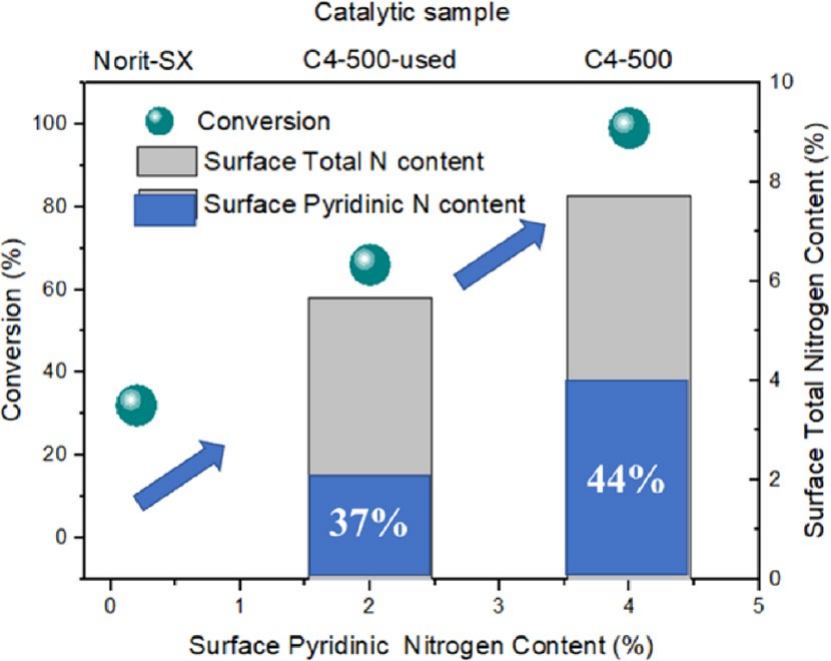
Correlation between catalytic activity and surface total and pyridinic nitrogen content.

Furthermore, in the N *1s* region, all three samples (C4-500, C5-500, and C4-500-used) displayed the presence of two bands around 399.0 and 400.4 eV, associated with the presence of pyridinic and pyrrolic species, respectively (Figure 7).^43^ XPS quantification analysis of the components in the N *1s* region of the samples C4-500 and C5-500 indicated similar results with a content of pyridinic nitrogen of (42 ± 1)% and a percentage of pyrrolic counterpart of (58 ± 1)%. This corroborated the observation that both materials had a similar catalytic behavior in the investigated process. Thought-provoking were the results obtained for the C4-500-used sample. Compared to the fresh parent catalyst, the recovered solid displayed a slight decrease and increase of the pyridinic and the pyrrolic content, respectively. This explains the loss of activity described in Figure 4, because pyrrolic sites are weaker basic sites than pyridine ones. In agreement with other literature reports,^25^ this confirms that the main contribution to the catalytic activity comes from the more basic pyridinic sites. This aspect was further clarified in Figure 9, where a direct relationship is highlighted between the surface pyridinic nitrogen content and the performance/activity of the investigated catalysts.

The O *1s* XPS region showed the presence of two main contributions around 531.6 eV and 533.3 eV, which were attributed to the O–O bond and to water adsorbed/bonded in the samples, respectively (Figure 8c). The presence of H_2_O could be coresponsible (along with the water content of EPI) for the formation of the diol 1b as a reaction byproduct.^42^

XPS quantification analyses of the observed regions are finally summarized in Table 4. It is worth highlighting that the C4-500 sample, with a surface nitrogen content of ca. 7.6%, exhibited a paramount catalytic activity, with conversion higher than 99%. A similar surface nitrogen content (ca. 6.4%) was found in the C5-500 material, which has been proposed as the main active center. However, in the case of the C5-500 sample, a concomitant (but not synergistic) effect of the calcium entities (ca. 6.2%) present in the material could not be ruled out.

**Table 4:** XPS Quantification Analysis of Representative Samples

sample	C *1s*	O *1s*	N *1s*	Cl *2p*	P *2p*	Ca *2p*	S *2p*
C4-500 fresh	81.68	10.58	7.66	0.07			
C4-500 used	76.39	17.47	5.73	0.40			
C5-600	57.94	26.81	6.41	0.29	1.97	6.19	0.41

The comparison of fresh and used C4-500 clearly showed that after the reaction, both a decrease in the nitrogen content and an increase in the oxygen and chlorine amount on the catalytic surface occurred. Overall, the results were consistent with the above-proposed hypothesis of adsorption of organic species (epoxide, diol, and cyclic carbonate products) on the basic pyridinic centers on the catalyst surface.

### Substrate Scope

**Scheme 3:**
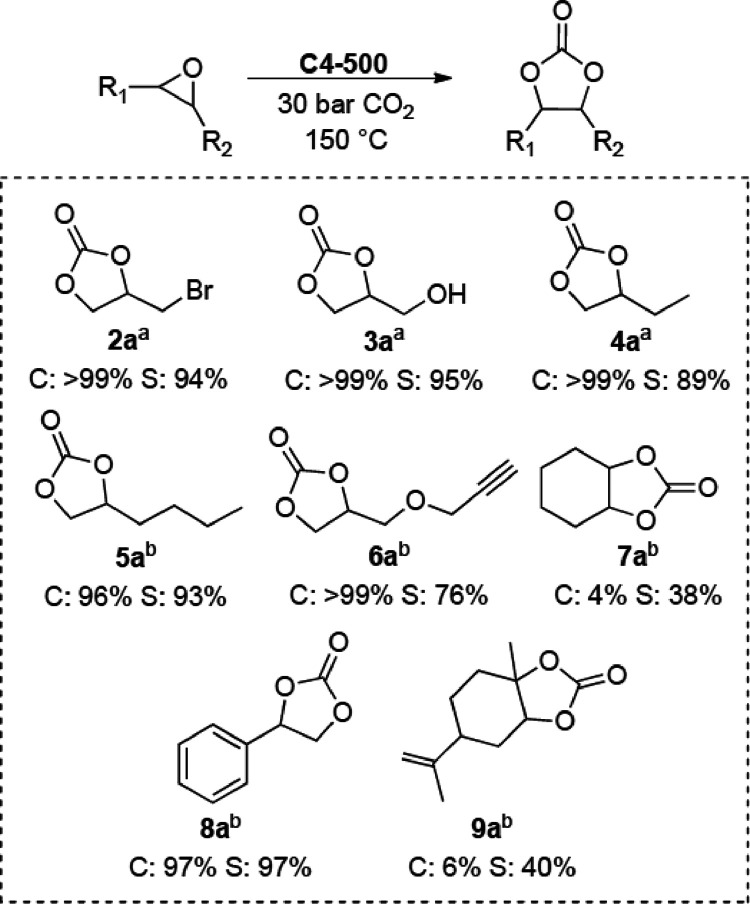
Substrate Scope and Epoxide (20 mmol), C4-500 (100 mg), 150 °C, 30 bar CO_2_ Reaction time: 4 h. Reaction time: 15 h. C = conversion %, S = selectivity %.

The model *N*-doped carbon C4-500 was tested to explore the cycloaddition of CO_2_ on other epoxides, both terminal and internal ones, than EPI. To the scope, epibromohydrin (2), glycidol (3), 1,2-epoxybutane (4), 1,2-epoxyhexane (5), cyclohexene oxide (6), styrene oxide (7), glycidyl propargyl ether (8), and limonene oxide (9) were used. A mixture of the chosen epoxide (20 mmol) and C4-500 (100 mg) was set to react at 150 °C under 30 bar of CO_2_. Experiments were carried out either for 4 h in analogy to conditions of Figure 4, or when necessary, prolonged for 15 h to offset changes of reactivity because of the intrinsic nature of the different epoxides (further details on this aspect are shown in Tables S2–S4 of the ESI section). Results are reported in Scheme 3 where superscripts a and b refer to reactions run for 4 and 15 h, respectively. Conversion and selectivity were determined by GC.

The batch protocol implemented for EPI proved of general applicability for terminal epoxides (2–6) including bifunctional molecules as epibromohydrin, glycidol and glycidyl propargyl ether, and styrene oxide 8. All the tested substrates allowed a very high conversion (96–99%) with good-to-excellent selectivity (76–99%) toward the corresponding carbonate products. On the other hand, internal epoxides 8 and 9 were scantly reactive: the cycloaddition proceeded with almost negligible conversion (4–6%) and carbonate selectivity not exceeding 40%. This behavior was largely anticipated by results already reported in the literature that impute the lack of reactivity of internal epoxides to steric hindrance effects.^44^

### CF Experiments

**Figure 10:**
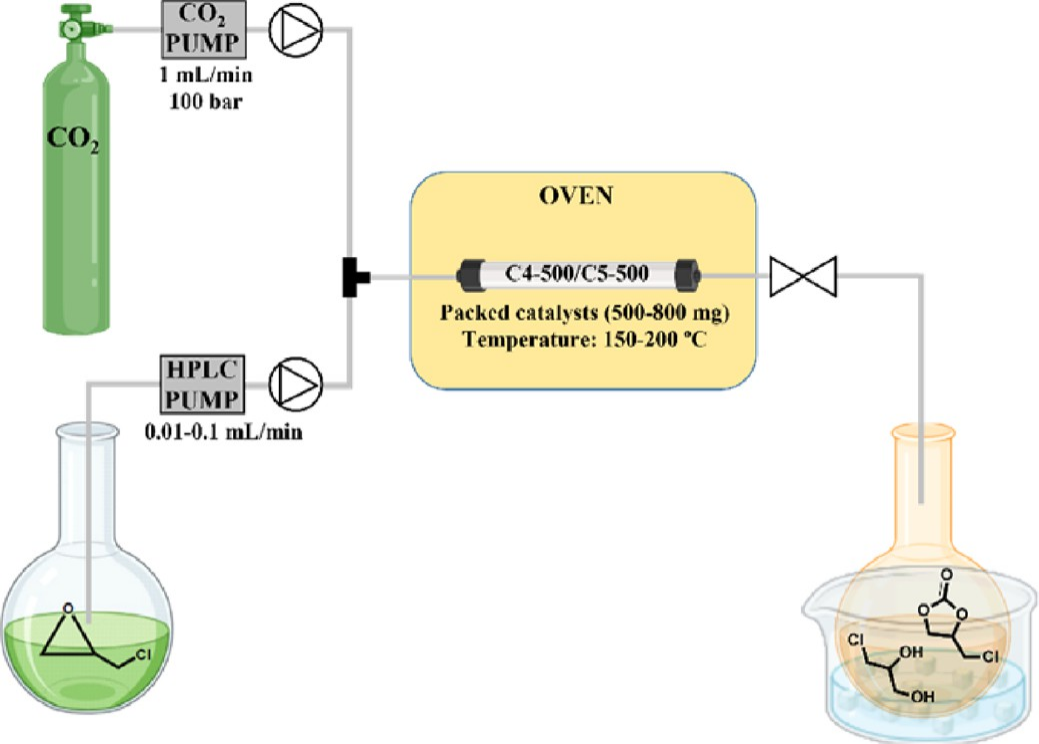
Lab setup designed for the CF synthesis of 4-(chloromethyl)-1,3-dioxolan-2-one (1a) from epichlorohydrin and CO_2_ in the presence of C4-500 and C5-500 catalysts.

The insertion of CO_2_ into EPI was selected as a model reaction to study the feasibility of the process in the CF mode. CF protocols are among the best and most reliable options not only to control/optimize the reaction parameters, but also to scale-up organic transformations and improve process intensification.^45^ In this context, also our group recently reported a continuous synthesis of cyclic carbonates from epoxides and CO_2_, catalyzed by a binary homogeneous mixture composed of diethylene glycol and NaBr.^32^ These results prompted us to extend the investigation to other heterogeneous catalysts as those studied here, with emphasis on both C4-500 and C5-500 samples. The flow apparatus was an in-house assembled system as schematized in Figure 10.

Experiments were carried out by exploring the effects of the temperature (*T*) and the flow rate (*F*) in the range of 150–200 °C and 0.01–0.1 mL min^–1^, respectively. The latter, however, was changed only for the liquid epoxide, while the flow of CO_2_ was set to a constant value of 1 mL/min consistent with the specs of the CO_2_ pump available in our CF system (Figure 9). Also, the operating pressure was set to 100 bar and was never modified throughout the study. Initial tests were performed by feeding a solution of epichlorohydrin in acetonitrile (ACN, 0.3 M) to a plug-flow reactor filled with the catalyst (C4-500 or C5-500: 600 mg). Then, further CF reactions were run using liquid EPI as such (neat). At the outlet of the reactor, the mixture was condensed into an ice bath, and at intervals, an aliquot was analyzed by GC and GC/MS to determine both conversion and selectivity and confirm the product structure. All reactions were prolonged for at least 3 h until the composition of the effluent mixture was steady over time, with variations within ±5% between one analysis and the other. The reaction productivity (*P*) defined as the amount (mmol) of carbonate produced per hour and mass unit (g) of the catalyst was also calculated in all cases. Results are reported in Table 5.

**Table 5:** CF Synthesis of Carbonate 1a from EPI and CO_2_^a^

entry	catalyst	*T* (°C)	EPI^b^ (mol L^–1^)	*F*^c^ (mL min^–1^)	conv. (%)	sel. 1a (%)	productivity 1a (mmol g^–1^ h^–1^)
1	C4-500	200	0.3	0.01	>99	95	0.4
2	C4-500	175	0.3	0.01	>99	97	0.4
3	C4-500	150	0.3	0.01	>99	99	0.5
4	C4-500	200	0.3	0.1	48	87	1.9
5	C4-500	150	neat	0.01	75	95	10.5
6	C5-500	150	0.3	0.01	>99	99	0.5
7	C5-500	150	neat	0.01	>99	99	15.0

a CO_2_ flow rate was 1 mL min^–1^ and the pressure was 100 bar in all cases.

b EPI fed in an acetonitrile solution (0.3 M) or as such (neat).

c *F* = flow rate of EPI (neat or in solution). Conversion of EPI and selectivity toward the carbonate 1a were determined by GC-FID analyses.

In the presence of C4-500, the first test run at 200 °C and *F* = 0.01 mL min^–1^ was encouraging. The reaction of a solution of EPI in ACN proceeded with complete conversion and 95% selectivity (entry 1). Even better results, however, were gathered without changing the conditions except for decreasing *T* to 175 and 150 °C: both processes were quantitative and the exclusive formation of 1a (up to 99%) was achieved with a productivity in the range of 0.4–0.5 mmol of 1a g_cat_^–1^ h^–1^ (entries 2 and 3). With the aim of improving the process efficiency, additional experiments were run by increasing either *F* up to a factor of 10 (0.1 mL min^–1^) and the EPI concentration until the solvent (ACN) was totally removed and the substrate was used neat. The two most representative results of this screening/optimization study are summarized in entries 4 and 5. At 200 °C, when *F* was gradually enhanced to 0.1 mL min^–1^, both the conversion and the selectivity dropped to 48 and 87%, respectively, but *P* was almost quadrupled (entry 4: 1.9 mmol g_cat_^–1^ h^–1^) compared to previous tests. An unexpectedly good outcome was achieved by feeding the CF reactor with pure EPI at 150 °C and tuning *F* at 0.01 mL min^–1^: at a conversion of 75%, the selectivity was 95%, meaning that *P* was boosted to 10.5 mmol g_cat_^–1^ h^–1^ (entry 5).

**Figure 11:**
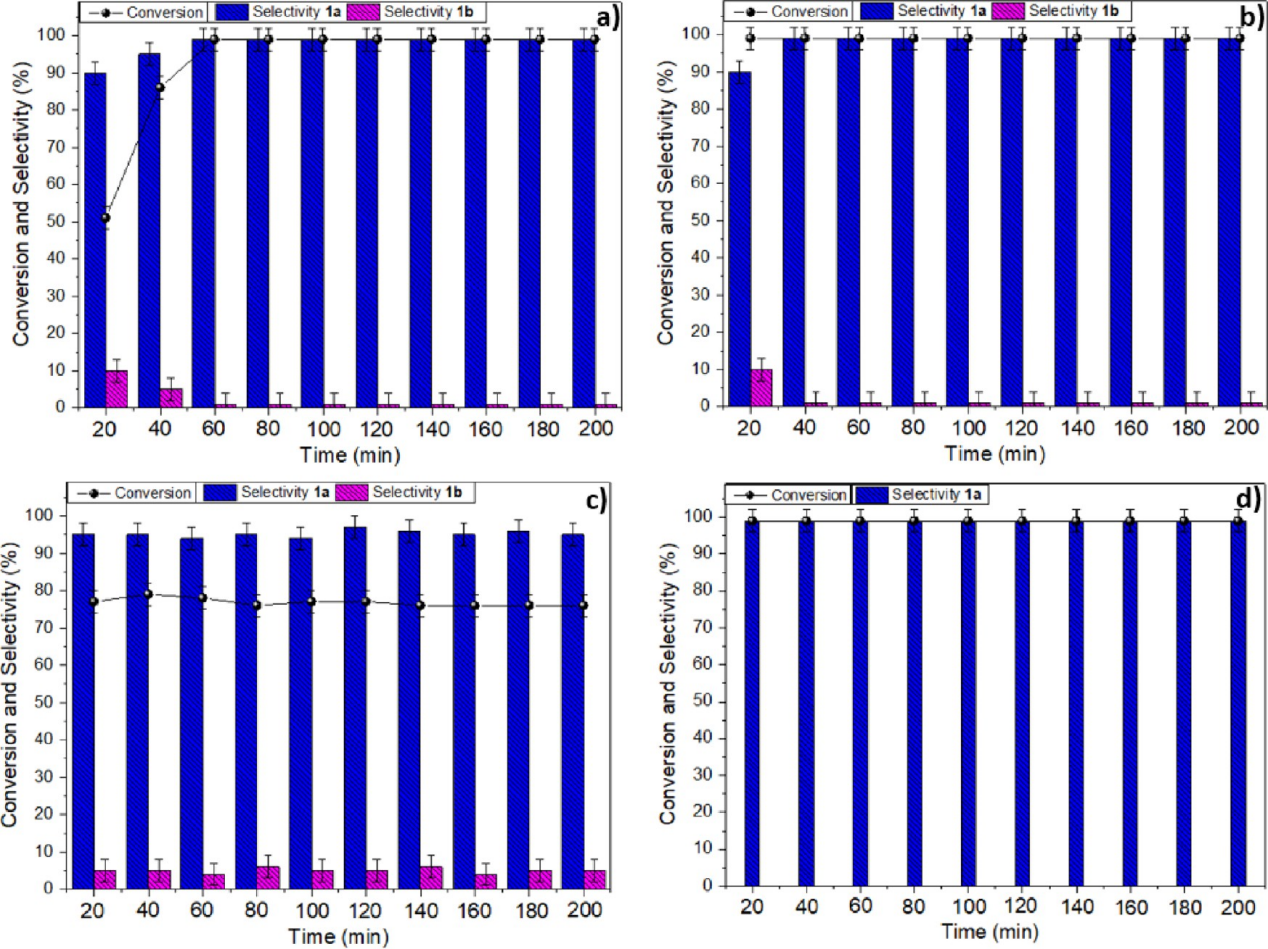
Catalytic performance of C4-500 and C5-500 samples in terms of conversion, selectivity, and on-stream stability in the CF cycloaddition of CO_2_ to epichlorohydrin. (A) C4-500, EPI solution in ACN (0.3 M); (B) C5-500, EPI solution in ACN (0.3 M); (C) C4-500, neat EPI; (D) C5-500, neat EPI.

In the presence of C5-500, results were equally good to those obtained with C4-500 when the substrate was fed in solution. At 150 °C and *F* = 0.01 mL min^–1^, the cycloaddition was quantitative, and carbonate 1a was the sole product detected at the outlet of the reactor (entry 6). Interestingly, however, the C5-500 allowed keeping the same high conversion and selectivity (both >99%) even when EPI was used neat. A further improvement of *P* up to 15.0 mmol g_cat_^–1^ h^–1^ was therefore granted (entry 7). This behavior was confirmed when reactions of entries 3 and 4–7 were more carefully followed over time. The results are reported in Figure 11 that shows the composition of the reaction mixtures sampled at regular intervals of 20 min each, for a total period of 200 min.

Figure 11a,b refers to C4-500 and C5-500, respectively, as catalysts for the cycloaddition carried out at 150 °C, using a solution of EPI in ACN. A difference between the two processes was appreciated in the initial part of experiments where the formation of the side-product 1b up to a 10% maximum was noticed after 40 and 20 min with C4-500 and C5-500, respectively. Then, a stationary state was reached, and the outcome of the two reactions was substantially overlapping with each other until the tests were concluded (200 min). The conversion and the carbonate selectivity were steady at >99% in both cases.

Figure 11c,d refers to the same couple of catalysts and conditions (C4-500 and C5-500; 150 °C), except that neat EPI was used. The two trends were remarkably different for the whole duration of the tests. Albeit the composition of the reaction mixtures was stable over time with both catalysts, as mentioned above (Table 5), the reaction catalyzed by C4-500 showed a conversion (ca. 77%) and 1a selectivity (ca. 95%) systematically lower than those achieved with C5-500. The latter proved, instead, outstanding in terms of on-stream reliability and performance (Figure 11d).

A comment should be placed here on the side-product 1b originated by a hydrolytic ring-opening of the starting epoxide. The decrease of this derivative shown in Figure 11a,b was consistent with a gradual elimination of water physiosorbed on the catalyst that was removed by the continuous delivery of a fresh reactant solution. By contrast, however, the amount of 1b (ca. 5%) remained almost constant throughout all the experiments of Figure 11c when neat EPI was used, thereby suggesting that water was continuously fed along with the reagent. This apparent dichotomy corroborated the hypothesis that water was plausibly supplied by both the epoxide and the catalyst in different modes and amounts, the first one (reagent) as a continuous source during the reaction, and the latter (catalyst) as a temporary source at the beginning of the process. Yet, the reasons why the diol 1b was not detected at all in the reaction of Figure 11d remain to be clarified.

The most striking and significant evidence was the stable performance of both C4-500 and C5-500 under CF conditions compared to batch reactions where recycling tests and characterization analyses of the same *N*-doped carbon materials indicated that they deactivated with use. Additional CF experiments—not described here—showed that reactions prolonged for 8 h proceeded with unchanged conversion and selectivity with respect to those of Figure 11. We postulated that the continuous feeding of a fresh reagent (as such or in solution) along with dense (supercritical) CO_2_ and the continuous removal of products helped to keep the catalytic surface clean and to stabilize the catalyst performance. No further tests were performed to verify such a hypothesis.

## Conclusions

The here reported work has been aimed at achieving multiple objectives, from the design of a simple and sustainable method to obtained metal-free *N*-doped carbons to the use of such solids as catalysts for the activation and incorporation of CO_2_ in the synthesis of durable chemicals/materials, and, finally, to the implementation of CO_2_ cycloaddition reactions in a continuous mode to improve productivity and process intensification. The overall strategy has been successfully pursued. In particular: (i) a (small) library of catalysts with built-in basicity has been prepared from chitosan, chitin, and SS wastes through a thermal treatment carried out in the absence of additional reagents and solvents; (ii) a variety of batch experiments have demonstrated that the *N*-doped carbons, mostly those derived from chitin and SSs, were excellent catalysts for the cycloaddition of CO_2_ to internal epoxides with quantitative conversion and selectivity to cyclic carbonate products up to 95–97%; and (iii) albeit investigated only for the model case of epichlorohydrin, the setup of a continuous solvent-free protocol for the CO_2_ cycloaddition has confirmed that the same *N*-doped carbons allowed not only to scale-up the reaction with a productivity up to 15.0 mmol g_cat_^–1^ h^–1^, but they were outstandingly stable materials that did not undergo deactivation over time, at least in the explored time frame (8 h). Compared to the very recent literature of the sector,^46,47^ the CF procedure proved to be a robust and competitive option for the synthesis of cyclic organic carbonates. A further optimization, however, will be the object of future studies to improve some engineering aspects through an upgraded CF apparatus to control the CO_2_ flow and the effect of pressure.

## Supporting Information Available

Additional experiments (Tables S1–S4) and characterization data: GC–MS and NMR spectra of the obtained products (PDF)
