# Supplementary material for: Metal-Free N-Doped Carbons for Solvent-Less CO2 Fixation Reactions: A Shrimp Shell Valorization Opportunity
Source: ACS Sustain Chem Eng. 2022 Sep 29;10(41):13835–48. doi: 10.1021/acssuschemeng.2c04443 (PMC9942530; doi:10.1021/acssuschemeng.2c04443)
Supplement: Supplementary file 1 — sc2c04443_si_002.pdf [file sc2c04443_si_002.pdf]

Metal-free *N*-doped carbons for solvent-less CO_2 fixation reactions: a shrimp shell valorization opportunity

Daniele Polidoro,[1] Alvise Perosa,[1] Enrique Rodríguez-Castellón,[2] Patrizia Canton,[1] Lidia Castoldi,[3] Daily Rodríguez-Padrón,[1,] Maurizio Selva[1]**

[1] Dipartimento di Scienze Molecolari e Nanosistemi, UniversitàCa' Foscari di Venezia, 30123 Venezia, Italy

[2] Department of Inorganic Chemistry, Facultad de Ciencias, Universidad de Málaga, Campus de Teatinos s/n, 29071 Málaga, Spain

[3] Laboratory of Catalysis and Catalytic Processes, Dipartimento di Energia, Politecnico di Milano, Via La Masa 34, 20156 Milano, Italy

Number of pages: 12;

Number of figures: 14;

Number of tables: 4

Index

TableS1. Effect of CO_2 Presence **S3**

TableS2. Substrate Scope over C4-500 at 4h. **S3**

TableS3. Substrate Scope over C4-500 at 15h **S4**

TableS4. Substrate Scope over Chitin at 15h **S5**

CHARACTERIZATION DATA **S6**

TableS1. Effect of CO_2 Presence

Entry	Catalyst	Conversion (%)[b]		Selectivity (%)[b]	
				1a	1b
1	C1-500	43		5	95
2	C2-500	33		3	97
3	C3-500	27		4	96
4	C4-500	25		5	95
5	Blank	1		nd.	nd.

Reaction conditions: Epichlorohydrine (10 mmol, 925 mg), Catalyst (50 mg), 150 °C, 15h; [b] Conversion and Selectivity were determined by GC; nd: not determined.

TableS2. Substrate Scope over C4-500 at 4h.

Entry	Epoxide	Conversion (%)[b]	Selectivity (%)[b]
1	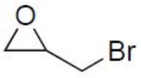 **2**	>99	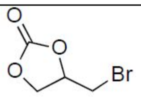 **2a**, 94%
2	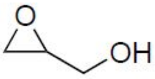 **3**	>99	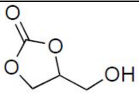 **3a,** 95%
3	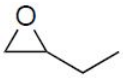 **4**	>99	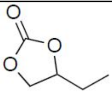 **4a,** 89%
4	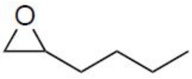 **5**	20	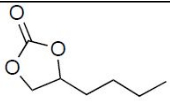 **5a,** 53%
5	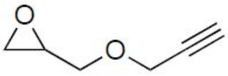 **6**	9	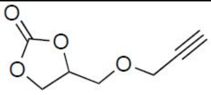 **6a,** 75%
6	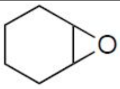 **7**	1	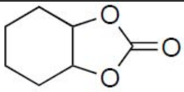 **7a,** 12%
7	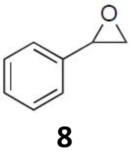 **8**	13	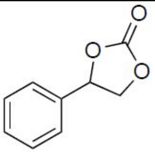 **8a,** 90%
8	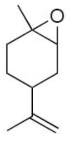 **9**	1	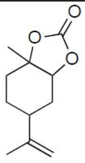 **9a,** 14%

Reaction conditions: Epoxide (10 mmol), C4-500 (50 mg), 150 °C, 30 bar CO_2, 4h; [b] Conversion and Selectivity were determined by GC

TableS3. Substrate Scope over C4-500 at 15h

Entry	Epoxide	Conversion (%)[b]	Selectivity (%)[b]
1	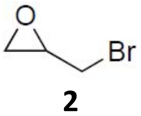 **2**	>99	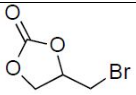 **2a**, 80%
2	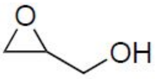 **3**	87	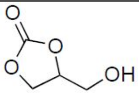 **3a,** 79%
3	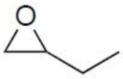 **4**	>99	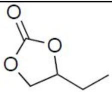 **4a,** 77%
4	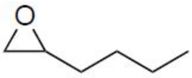 **5**	96	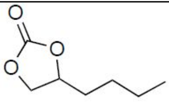 **5a,** 93%
5	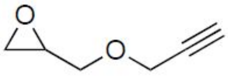 **6**	>99	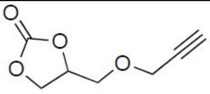 **6a,** 76%
6	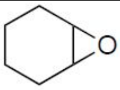 **7**	4	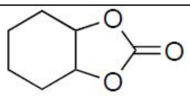 **7a,** 38%
7	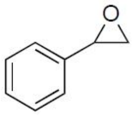 **8**	97	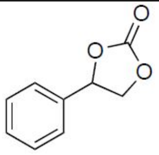 **8a,** 97%
8	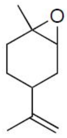 **9**	6	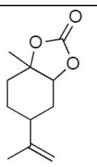 **9a,** 40%

Reaction conditions: Epoxide (10 mmol), C4-500 (50 mg), 150 °C, 30 bar CO_2, 15h; [b] Conversion and Selectivity were determined by GC

TableS4. Substrate Scope over Chitin at 15h

Entry	Epoxide	Conversion (%)[b]	Selectivity (%)[b]
1	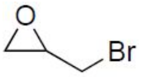 **2**	97	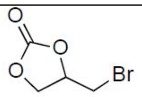 **2a**, 71%
2	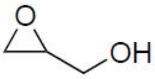 **3**	98	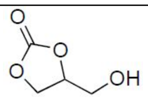 **3a,** 62%
3	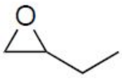 **4**	96	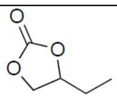 **4a,** 58%
4	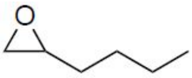 **5**	30	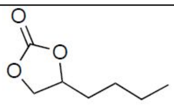 **5a,** 90%
7	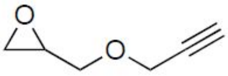 **6**	90	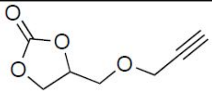 **6a,** 75%
5	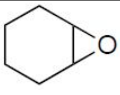 **7**	1	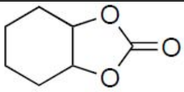 **7a,** 7%
6	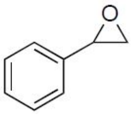 **8**	10	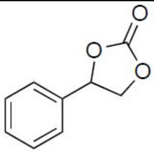 **8a,** 80%
8	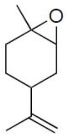 **9**	1	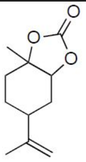 **9a,** 9%

Reaction conditions: Epoxide (10 mmol), Chitin (50 mg), 150 °C, 30 bar CO_2, 15h; [b] Conversion and Selectivity were determined by GC.

CHARACTERIZATION DATA

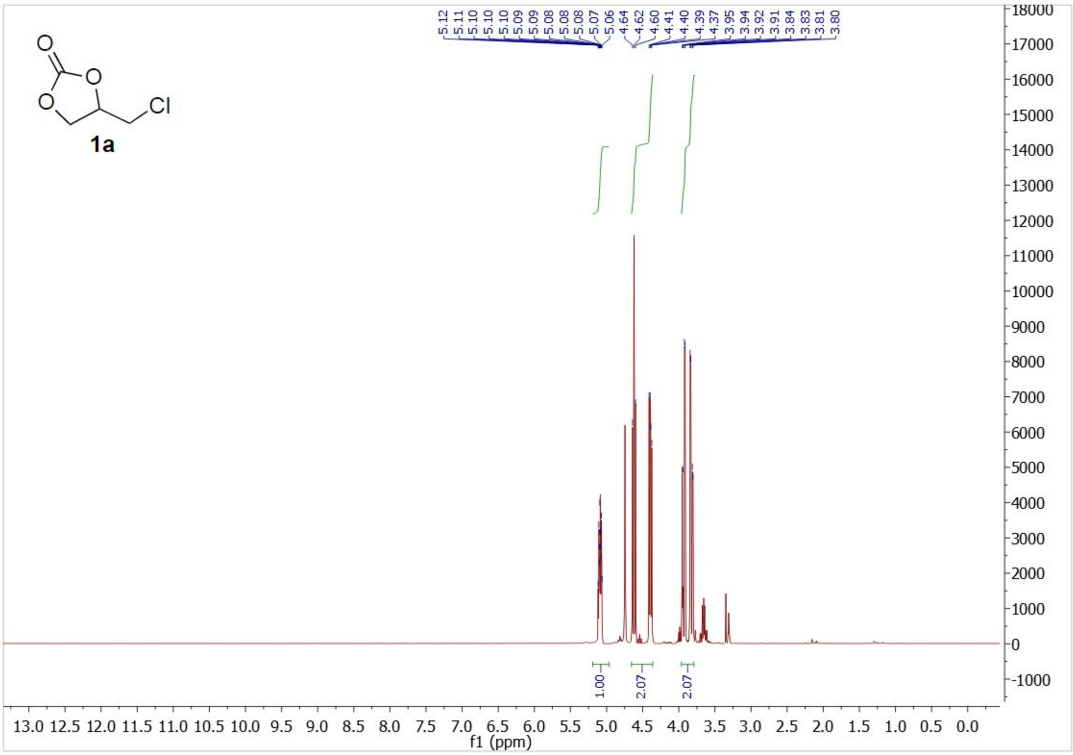

Figure S1. ^{1}H NMR (400 MHz, 298 K, MeOD) of **1a**.

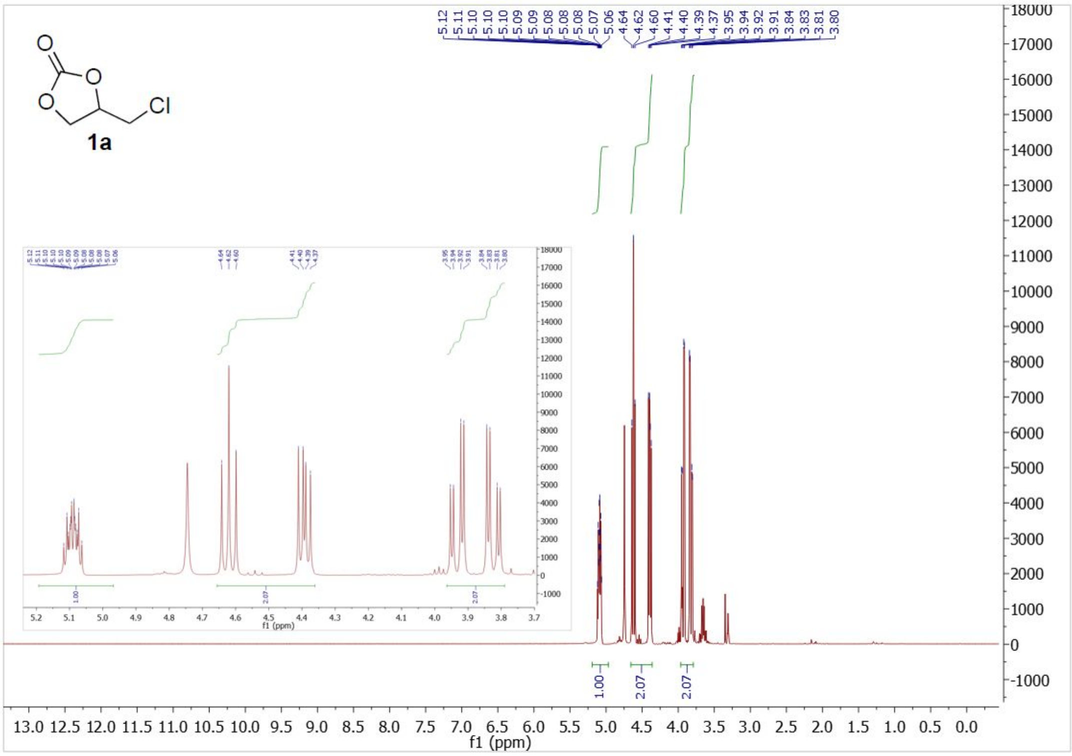

Figure S2. ^{1}H NMR (400 MHz, 298 K, MeOD) of **1a**.

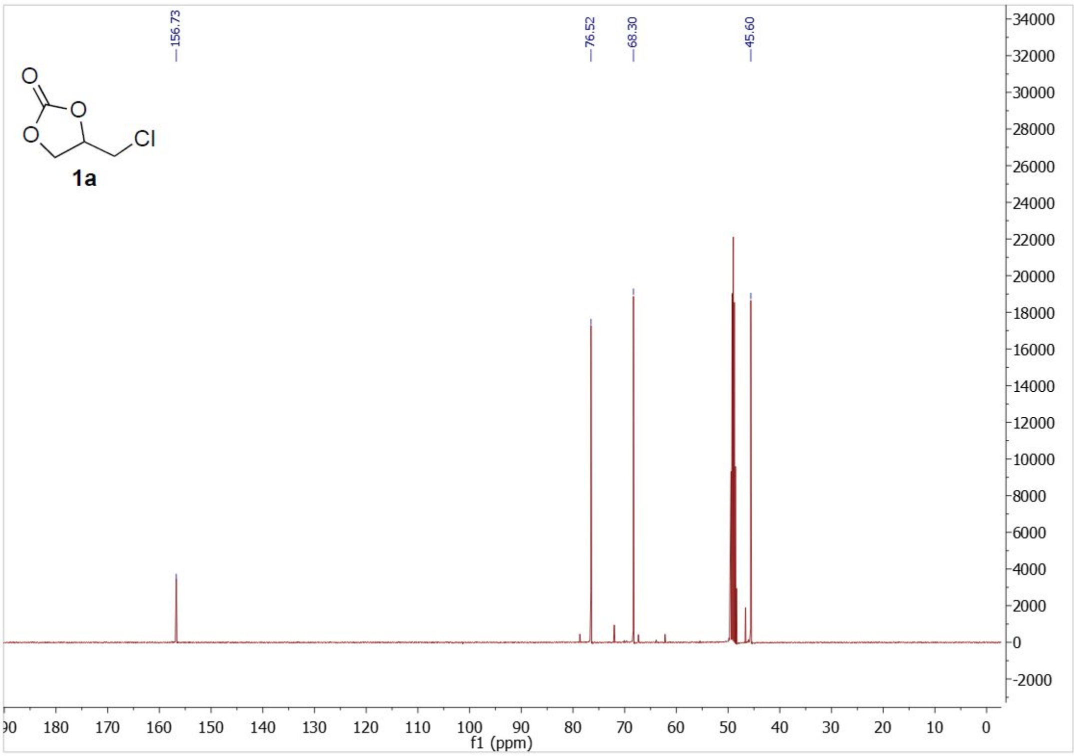

Figure S3. ^{13}C NMR (101 MHz, 298 K, MeOD) of **1a**.

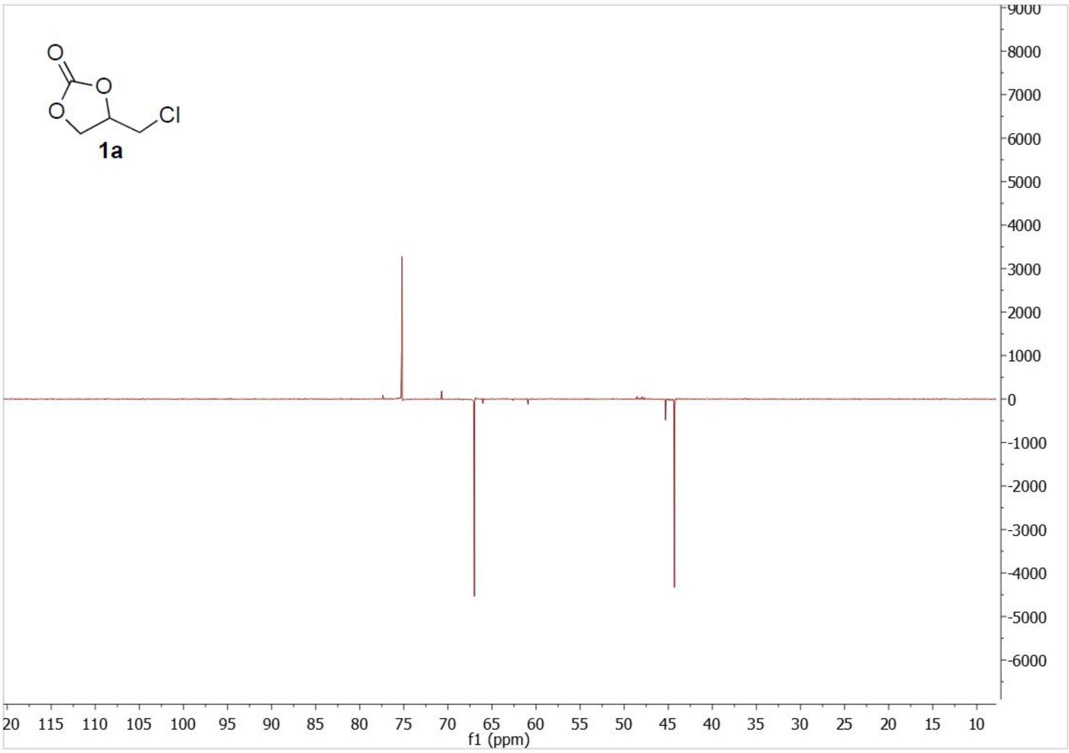

Figure S4. DEPT-135 (400 MHz, 298 K, MeOD) of **1a**.

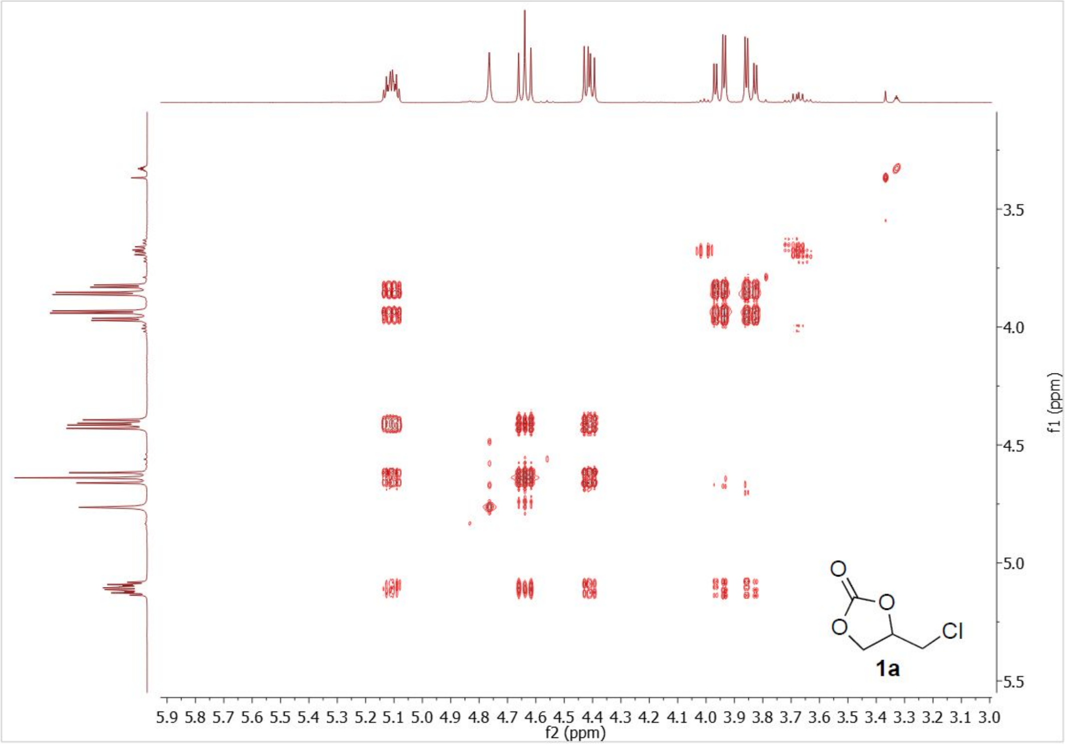

Figure S5. COSY (400 MHz, 298 K, MeOD) of **1a**.

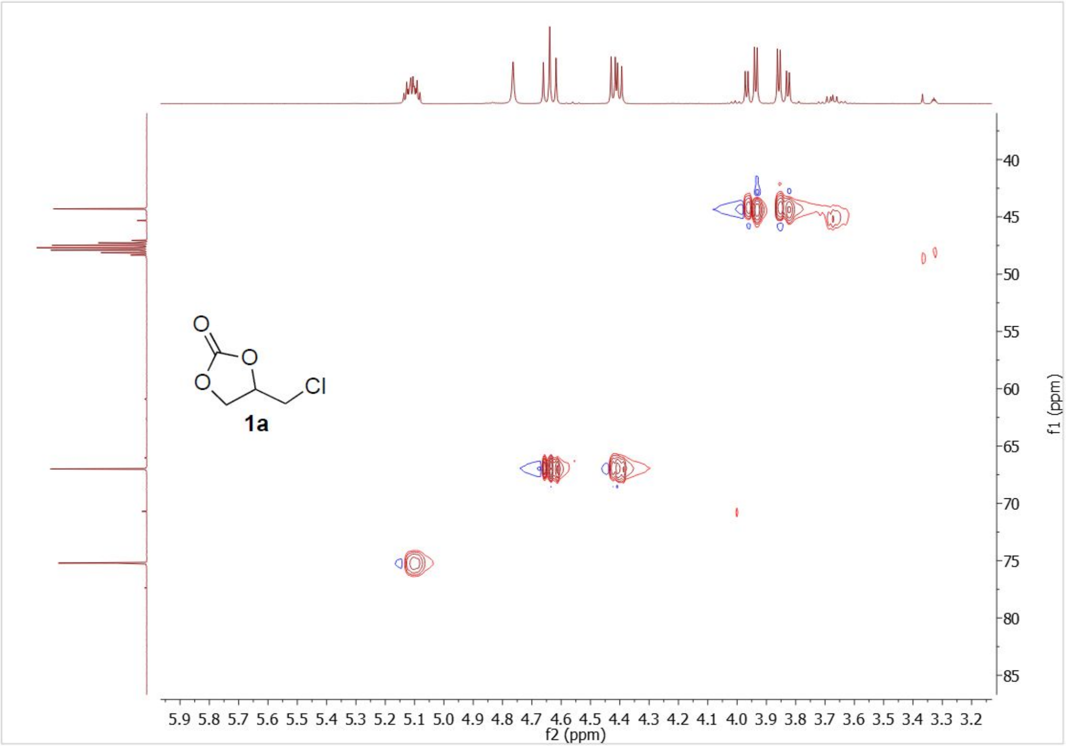

Figure S6. HSQC (400 MHz, 298 K, MeOD) of **1a**.

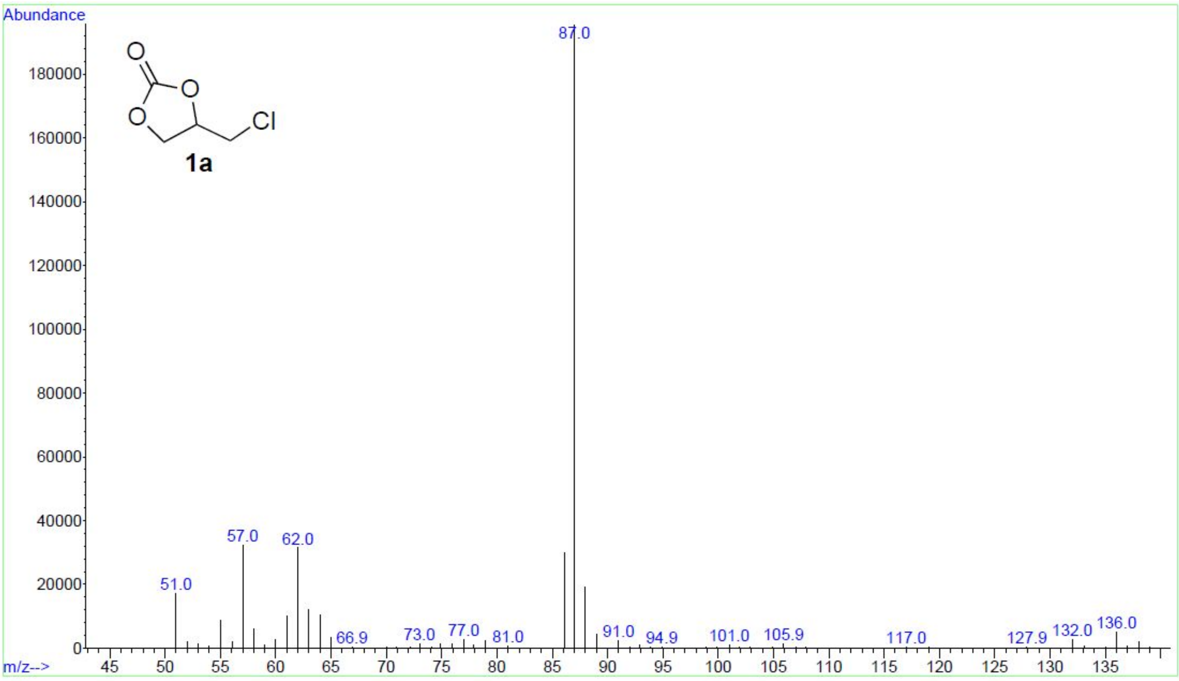

Figure S7. Mass Spectrum of **1a** (EI, 70 eV).

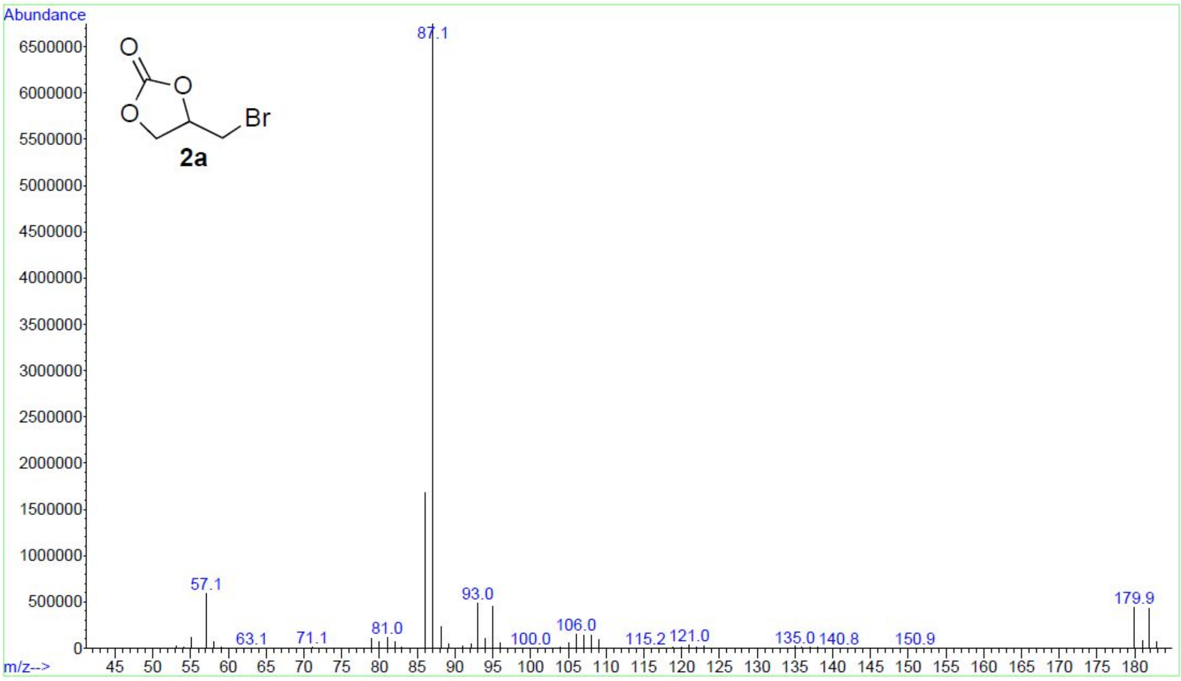

Figure S8. Mass Spectrum of **2a** (EI, 70 eV).

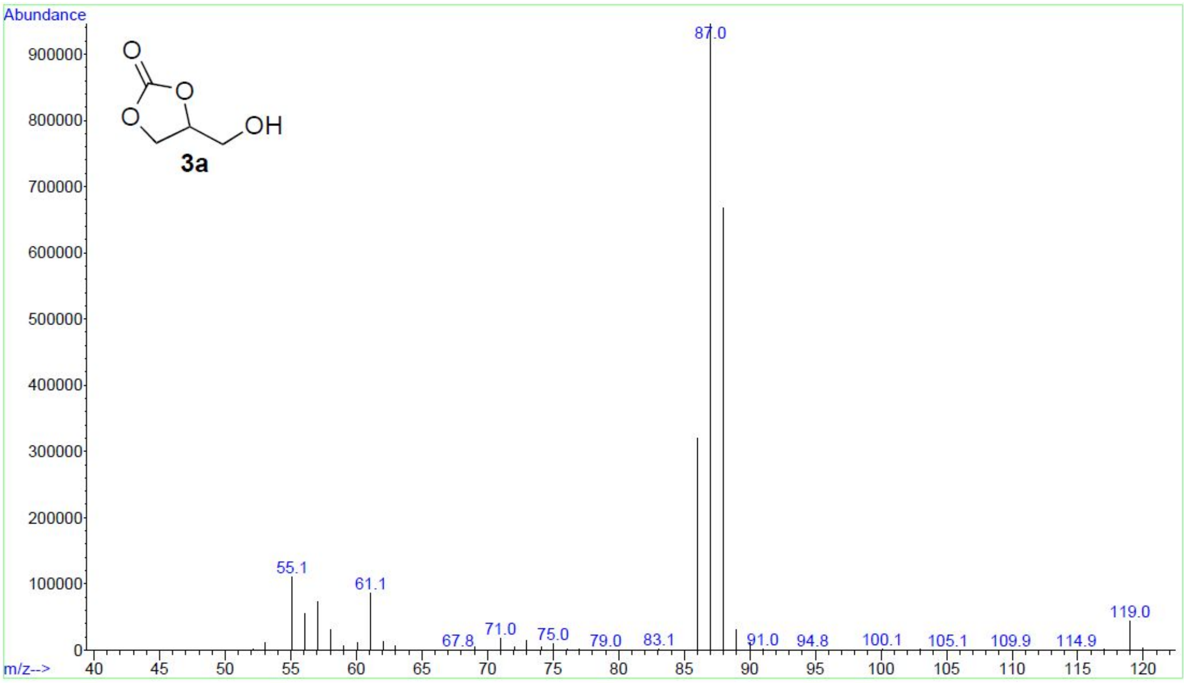

Figure S9. Mass Spectrum of **3a** (EI, 70 eV).

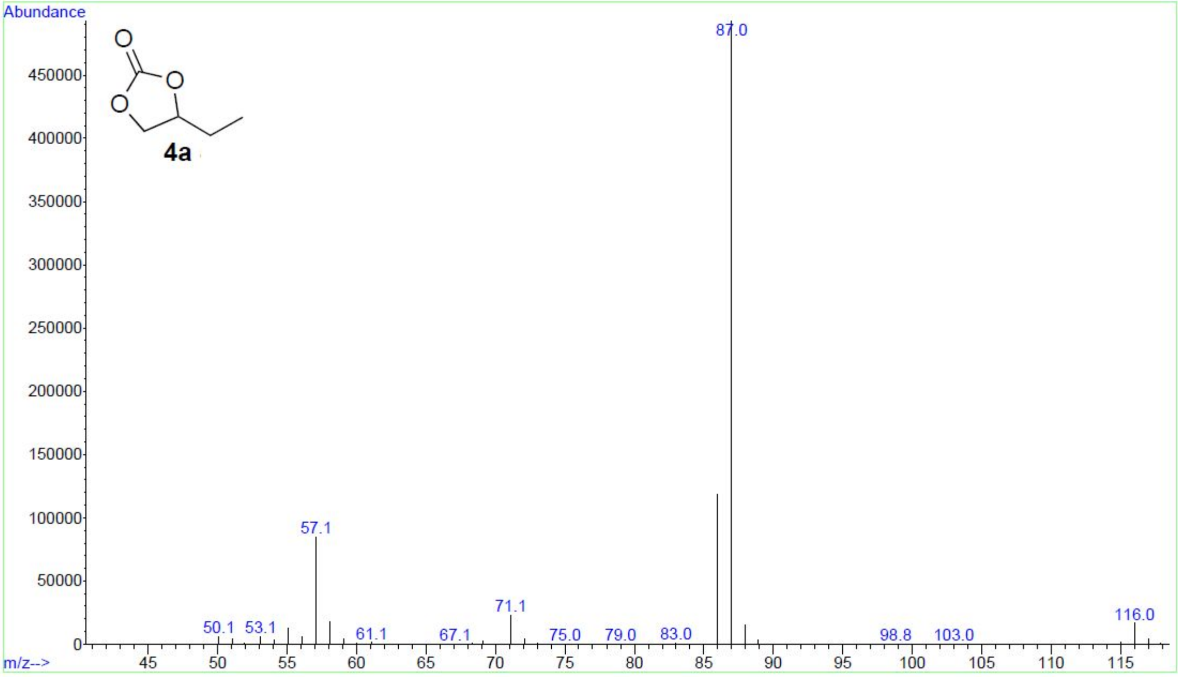

Figure S10. Mass Spectrum of **4a** (EI, 70 eV).

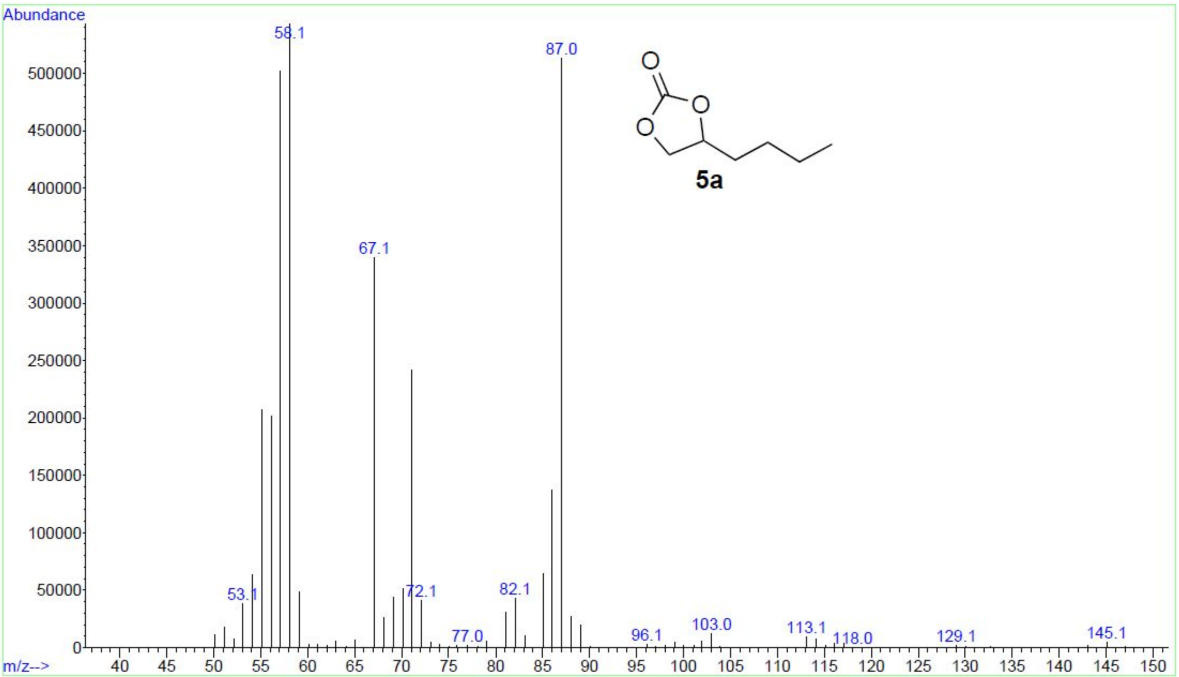

Figure S11. Mass Spectrum of **5a** (EI, 70 eV).

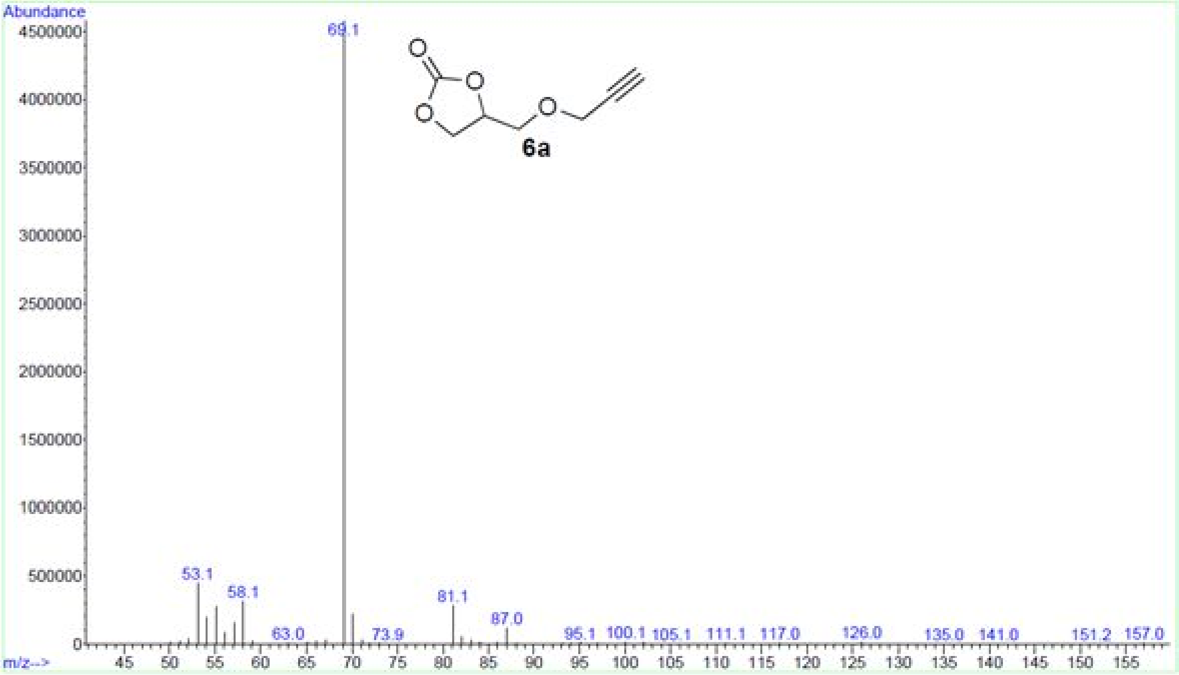

Figure S12. Mass Spectrum of **6a** (EI, 70 eV).

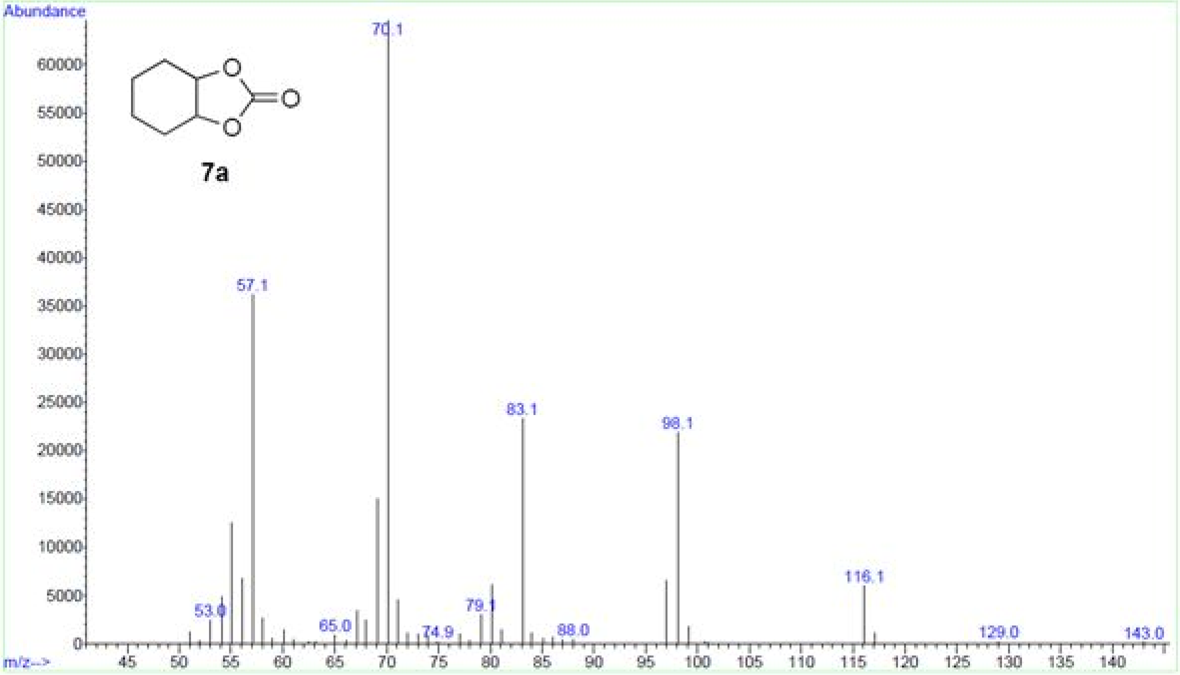

Figure S13. Mass Spectrum of **7a** (EI, 70 eV).

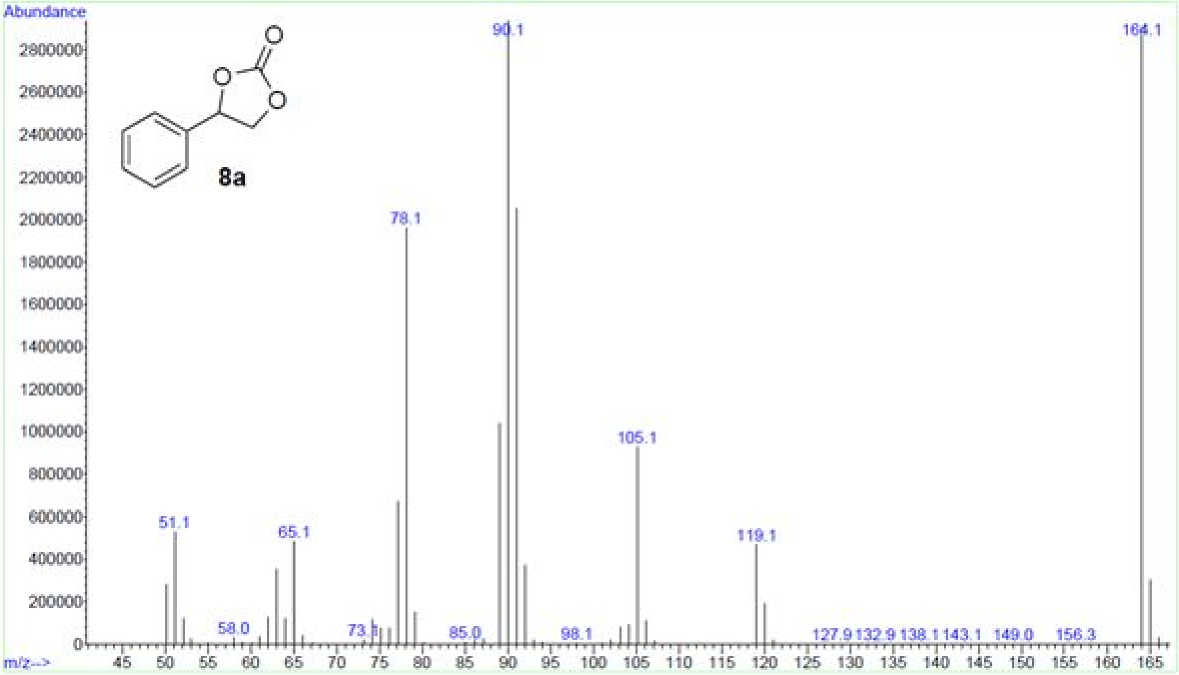

Figure S14. Mass Spectrum of **8a** (EI, 70 eV).
